# Sox5 Functions as a Fate Switch in Medaka Pigment Cell Development

**DOI:** 10.1371/journal.pgen.1004246

**Published:** 2014-04-03

**Authors:** Yusuke Nagao, Takao Suzuki, Atsushi Shimizu, Tetsuaki Kimura, Ryoko Seki, Tomoko Adachi, Chikako Inoue, Yoshihiro Omae, Yasuhiro Kamei, Ikuyo Hara, Yoshihito Taniguchi, Kiyoshi Naruse, Yuko Wakamatsu, Robert N. Kelsh, Masahiko Hibi, Hisashi Hashimoto

**Affiliations:** 1 Division of Biological Science, Graduate School of Science, Nagoya University, Furo-cho, Chikusa-ku, Nagoya, Aichi, Japan; 2 Division of Biomedical Information Analysis, Iwate Tohoku Medical Megabank Organization, Iwate Medical University, Yahaba-cho, Shiwa-gun, Iwate, Japan; 3 National Institute for Basic Biology, Interuniversity Bio-Backup Project Center, Okazaki, Aichi, Japan; 4 Department of Basic Biology, School of Life Science, The Graduate University for Advanced Studies (SOKENDAI), Myodaiji, Okazaki, Aichi, Japan; 5 Bioscience and Biotechnology Center, Nagoya University, Furo-cho, Chikusa-ku, Nagoya, Aichi, Japan; 6 Centre for Regenerative Medicine and Department of Biology and Biochemistry, University of Bath, Bath, Claverton Down, United Kingdom; 7 Spectrography and Bioimaging Facility, National Institute for Basic Biology, Myodaiji, Okazaki, Aichi, Japan; 8 Laboratory of Bioresources, National Institute for Basic Biology, Myodaiji, Okazaki, Aichi, Japan; 9 Department of Preventive Medicine and Public Health, School of Medicine, Keio University, Shinjuku-ku, Tokyo, Japan; University of Washington, United States of America

## Abstract

Mechanisms generating diverse cell types from multipotent progenitors are crucial for normal development. Neural crest cells (NCCs) are multipotent stem cells that give rise to numerous cell-types, including pigment cells. Medaka has four types of NCC-derived pigment cells (xanthophores, leucophores, melanophores and iridophores), making medaka pigment cell development an excellent model for studying the mechanisms controlling specification of distinct cell types from a multipotent progenitor. Medaka *many leucophores-3 (ml-3)* mutant embryos exhibit a unique phenotype characterized by excessive formation of leucophores and absence of xanthophores. We show that *ml-3* encodes *sox5*, which is expressed in premigratory NCCs and differentiating xanthophores. Cell transplantation studies reveal a cell-autonomous role of *sox5* in the xanthophore lineage. *pax7a* is expressed in NCCs and required for both xanthophore and leucophore lineages; we demonstrate that Sox5 functions downstream of Pax7a. We propose a model in which multipotent NCCs first give rise to *pax7a*-positive partially fate-restricted intermediate progenitors for xanthophores and leucophores; some of these progenitors then express *sox5*, and as a result of Sox5 action develop into xanthophores. Our results provide the first demonstration that Sox5 can function as a molecular switch driving specification of a specific cell-fate (xanthophore) from a partially-restricted, but still multipotent, progenitor (the shared xanthophore-leucophore progenitor).

## Author Summary

How individual cell fates are specified from multipotent progenitor cells is a fundamental question in developmental and stem cell biology. Accumulating evidence indicates that stem cells develop into each of their final, diverse cell-types after progression through one or more partially-restricted intermediates, but the molecular mechanisms underlying final fate choice are largely unknown. Neural crest cells (NCCs) give rise to diverse cell-types including multiple pigment cells and thus are a favored model for understanding the mechanism of fate specification. We have investigated how a specific fate choice is made from partially-restricted pigment cell progenitors in medaka. We show that Sry-related transcription factor Sox5 is required for fate determination between yellow xanthophore and white leucophore, and its loss causes excessive formation of leucophores and absence of xanthophores. We demonstrate that Sox5 functions cell-autonomously in the xanthophore lineage in medaka. Furthermore, *pax7a* is expressed in the partially-restricted progenitor cells shared with xanthophore and leucophore lineages, and Sox5 acts in some of these cells to promote xanthophore lineage. Our work reveals the role of Sox5 as a molecular switch determining xanthophore versus leucophore fate choice from the shared progenitor, and identifies an important mechanism regulating pigment cell fate choice from NCCs.

## Introduction

Elucidation of mechanisms that control cell fate specification from multipotent progenitors is one of the most important topics in developmental biology. The neural crest is a vertebrate-specific multipotent cell population that arises at the border of the neural plate and prospective epidermis. The neural crest cells (NCCs) migrate to various destinations along stereotyped pathways and thereby give rise to a diversity of different cell types, including sensory, enteric and autonomic neurons, glia of the peripheral nervous system, skeletogenic fates such as craniofacial cartilage, and pigment cells [1], [2]. Their stem cell-like characteristics, and potential therapeutic uses in regenerative medicine, make NCCs attractive as a model for studying fate specification of multipotent progenitor cells in stem cell biology.

Neural crest-derived pigment cells in vertebrates are classified into diverse cell types and each can be easily identified by its natural coloration. Whereas mammals and birds have a single type of pigment cell, melanocytes, fish have up to six [3]. Zebrafish has three distinct types of pigment cells, melanophores, yellow xanthophores and iridescent iridophores [4], [5]. In medaka and some other fish species, there is a fourth pigment cell type, the white leucophore [6]–[8]. The white coloration and auto-fluorescence of leucophores depend on light reflection from intracellular organellar crystals of uric acid, which is a purine-related substance similar to guanine and hypoxanthine in iridophores [9]. In addition, leucophores in medaka embryos and larvae appear to be orange, due to production of a pteridine pigment, drosopterin, during embryonic/larval stages [10]. Xanthophores contain a different pteridine, yellow sepiapterin. Biochemical studies suggest that both drosopterin and sepiapterin are generated via a common synthesis pathway for H_4_biopterine from GTP [11], indicating a possible close evolutionary relationship between xanthophores and leucophores, although the embryological/genetic relationship between these two cell-types has not been studied.

Previous studies by in vitro clonal analysis showed that multipotent NCCs become gradually restricted in their potential to generate certain derivatives, forming partially-restricted intermediate progenitors before eventually becoming specified to an individual fate [12]–[14]. However, the presence, identity and diversity of these intermediate progenitors in vivo remains unclear. Furthermore, the molecular mechanisms regulating fate specification from the intermediate progenitors are also incompletely understood. Previous genetic studies identified a few key transcription factors required for fate specification within NCCs [15]. Some of the best-known examples are transcription factors controlling development of melanocytes (functionally and genetically equivalent to melanophores in fish). Sox10 is required to drive transcription of *Mitf*, a master gene for melanocyte lineage in multipotent NCCs [16]–[21]. Pax3 acts synergistically with Sox10 to regulate the mouse *Mitf* promoter [22]–[25]. Whereas the molecular mechanisms driving melanocyte differentiation are relatively well understood, the equivalent mechanisms for other pigment cell-types remain largely mysterious, as do the interactions between these factors that control the balance of pigment cell fates. One exception comes from the demonstration in zebrafish and chick that Foxd3 functions to inhibit differentiation of melanocytes from NCCs by repressing *Mitf* function; in zebrafish, FoxD3 also promotes iridophore development [26]–[29].

The mutant collections in medaka provide models for investigating the genetic basis of fate choice in NCCs. A series of loci affecting both xanthophore and leucophore development in medaka offer novel insight into how these two pigment cell types are specified [30], [31]. Among them, *leucophore free* and *leucophore free-2* (*lf* and *lf-2*) mutants have no discernible leucophores nor xanthophores in the embryo/larva, although *lf-2* mutants have occasional escaper leucophores [31]. Our previous studies revealed that the *lf-2* locus encodes *pax7a*, and *lf-2* mutants failed to develop xanthophores and leucophores, suggesting a role of *pax7a* in fate specification of a shared, partially-restricted progenitor of the xanthophore and leucophore lineages (Kimura et al, unpublished data). We also revealed that *lf* locus is *slc2a15b* and this encodes a solute career (Slc) protein required for coloration of xanthophores and leucophores, consistent with the previous description that *lf* mutants have deficiently-colored xanthophores and leucophores. These genetic data also suggest that leucophores are closely related to xanthophores. This left the major unanswered question of what mechanism allows selection of xanthophore or leucophore fate from these intermediate progenitors.

Medaka *many leucophores-3 (ml-3)* mutant embryos exhibit excessive formation of leucophores and absence of visible xanthophores [30]–[32]. To elucidate the molecular mechanisms regulating the fate choices of xanthophore and leucophore, we have investigated medaka *ml-3* mutant by genetic and developmental approaches. Here we report that *ml-3* locus encodes medaka *sox5* and that Sox5 functions cell-autonomously to control specification of xanthophores from the shared xanthophore-leucophore progenitor.

## Results

### *ml-3* mutants show combined ectopic and excessive formation of leucophores and absence of xanthophores

**Figure 1:**
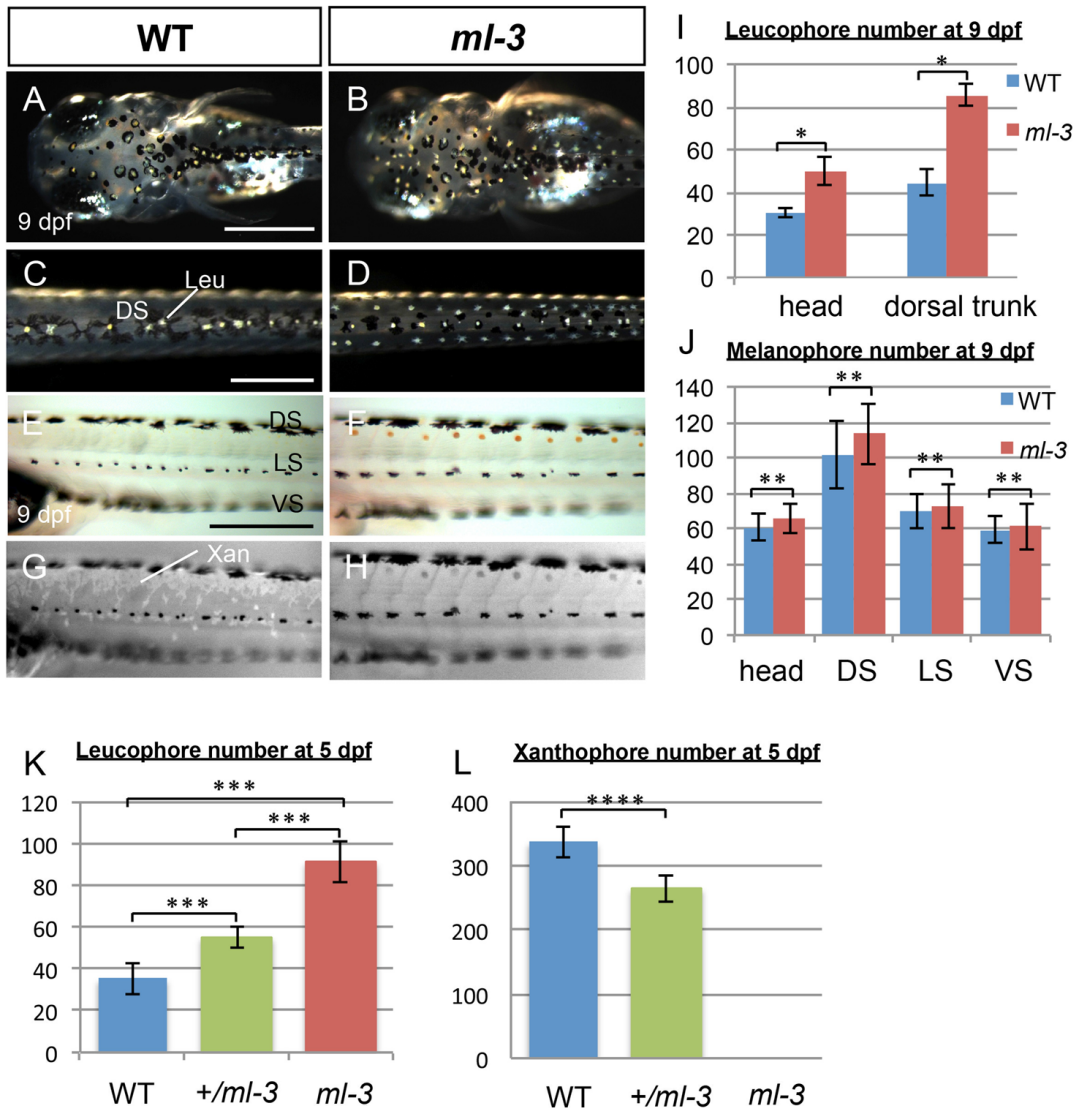
Larval pigment pattern phenotypes of medaka *ml-3* mutants. (A–H) 9 dpf (hatching stage). (A, C, E, G) WT. (B, D, F, H) *ml-3* mutant. (E, F) Bright field. (G, H) UV light. (A–D) Dorsal views. (E–H) Lateral views. (A–D) Leucophores are formed in excess in mutants. Whereas in WT larvae leucophores lie over the head (A) and along dorsal midline of the trunk (C) in association with melanophores, in *ml-3* larvae excess leucophores are localized ectopically to form bilateral lines along the anterior-posterior axis (B, D) in addition to those in the normal position in head (B) and trunk midline (D). (E–H) Xanthophores are absent from *ml-3* mutants. In WT (E) as well as in *ml-3* (F), xanthophores are not evident when observed in brightfield. Under UV illumination, however, auto-fluorescent xanthophores are readily visible in WT, covering over dorsal trunk (G). In *ml-3* mutants, xanthophore auto-fluorescence is not detected (H). (A–H) Melanophore patterns are unaffected in *ml-3* mutants. In both WT (E, G) and *ml-3* mutants (F, H), melanophores are distributed in three longitudinal stripes (DS, LS and VS). (I, J) Counts of leucophores and melanophores in *ml-3* mutants at 9 dpf. Mean counts (error bars give standard deviation) are shown for WT (blue) and *ml-3* mutants (red). (I) The numbers of leucophores in head region and in dorsal trunk are larger in *ml-3* than in WT (n = 12 for each group and region): two-way ANOVA, *F*(group) = 389.09, df = 1,42, *p*<0.0001; *F*(group×region) = 46.93, df = 1,42, *p*<0.0001 and Student's *t*-test for each region, *, *p*<0.0001. (J) There is no significant difference in melanophore number between WT and *ml-3* (n = 12 for WT, n = 11 for *ml-3*): two-way ANOVA, *F*(group) = 3.72, df = 1,88, *p*>0.05; *F*(group×region) = 0.74, df = 1,88, *p*>0.05 and Student's *t*-test for each region, **, *p*>0.05. (K, L) Mean (±s.d.) counts of leucophores and xanthophores on dorsal trunk surface in WT, *ml-3* heterozygotes (+/*ml-3*) and *ml-3* homozygotes (*ml-3*) at 5 dpf (also see Figures S2E–S2J). (K) The leucophore numbers seem to be negatively correlated with the copy-number of *ml-3* gene (n = 10 for each group). *p*<0.0001 by one-way ANOVA. ***, *p*<0.0001 by Student's *t*-test for WT versus *ml-3* heterozygotes, WT versus *ml-3* homozygotes, and *ml-3* heterozygotes versus homozygotes. (L) The xanthophore numbers seem to be positively correlated with the copy-number of *ml-3* gene (n = 10 for each group). *p*<0.0001 by one-way ANOVA. ****, *p*<0.0001 by Student's *t*-test. Leu, leucophore; Xan, xanthophore; DS, dorsal stripe; LS, lateral stripe; VS, ventral stripe. Scale bars: 250 µm.

Medaka *ml-3* is a spontaneous mutant with excessive formation of leucophores, and a lack of visible xanthophore pigmentation in the embryo/larva [30], [31], but with no apparent phenotypes of other neural crest derivatives (Figures S1A–S1J). We first re-examined all pigment cell types during embryonic development in *ml-3* mutant. In wild-type (WT) embryos, the first differentiating leucophores appear ventral to the hindbrain/midbrain (ventral head leucophores) by 18–19 somite stage (50 hours post fertilization: hpf, data not shown) [31], [33]. The other population of leucophores, found on the dorsal surface of the head and trunk, begin to differentiate at 3.5–4 days post fertilization (dpf) when a few are visible (Figure S2A). By hatching stage (9 dpf), these leucophores have increased to approximately 30 cells scattered on the dorsal head and 40 cells in the dorsal midline of the trunk (Figures 1A, 1C, 1I). In *ml-3* homozygous mutants, formation of ventral head leucophores was not markedly altered (data not shown); in contrast, there is already an excess compared with WT of leucophores in the dorsal head and trunk at 4 dpf, (Figures S2A, S2B). By 7 dpf, the trunk leucophores are scattered more laterally than in WT, forming three lines (Figures 1D, S2C, S2D). Subsequently, leucophores in the central line of the three become gradually reduced, the number in the more lateral lines become substantially increased after hatching. The number of leucophores in *ml-3* homozygotes was significantly larger in both head and dorsal trunk than that in WT (Figures 1I, S2P).

Pigmented xanthophores are first observed at 6 dpf in WT, but differentiating xanthophores are difficult to detect at early embryonic stages. In order to detect them more readily, we examined them using their auto-fluorescence upon UV-light exposure (Figures 1E–1H) [34]. In WT hatchlings, numerous xanthophores cover the dorsal trunk and others are scattered around the melanophores in the lateral stripe (see below, Figure 1G). In *ml-3* homozygotes, no xanthophores were detected on the body surface (Figure 1H).

To further elucidate the effect of *ml-3* deficiency, we investigated the phenotypes of *ml-3* heterozygotes. The *ml-3* heterozygous leucophores were normally positioned in the dorsal midline of the trunk at 5 dpf, but significantly increased, compared with WT leucophores (Figures 1K, S2E–S2G). Xanthophores, scattered over the trunk surface, were significantly fewer at 5 dpf in *ml-3* heterozygotes than in WT (Figures 1L, S2H–S2J). These results indicate that *ml-3* is a semi-dominant mutation and the increase of leucophores and the decrease of xanthophores are dependent on *ml-3* gene dosage. These data suggest that the *ml-3* locus acts as a fate-switch for xanthophore versus leucophore lineages within a shared progenitor cell. Further experiments below were carried out using *ml-3* homozygotes (described as *ml-3* mutants) unless otherwise specified.

We found no evidence for a change in the number of the other two pigment cell-types, melanophores or iridophores. Melanophores first appeared in the trunk region at 12 somite stage (41 hpf) and formed three stripes in trunk: dorsal stripe (DS), lateral stripe (LS), and ventral stripe (VS) (Figure 1E). Whereas most melanophores in WT were associated with leucophores in the DS and head region (Figures 1A, 1C), there are no melanophores associated with the ectopic leucophores in *ml-3* hatchlings (Figures 1B, 1D, 1F). Consistent with this, the number of melanophores was not significantly different between WT and *ml-3* in either of the DS, LS, VS or head region (Figures 1J, S2O). Iridophores appeared on the eyes and the yolk sac at 5 dpf and could be readily observed at later stages in WT (data not shown and Figures S2K, S2M). Although we were unable to count numbers of iridophores due to ambiguity in their cell borders, there was no marked difference in the areas covered by iridophores in WT and *ml-3* (Figures S2L, S2N). These findings indicate that the *ml-3* mutation specifically affects the formation of xanthophores and leucophores, but not of melanophores and iridophores.

### Xanthophore specification is defective in *ml-3* mutants

**Figure 2:**
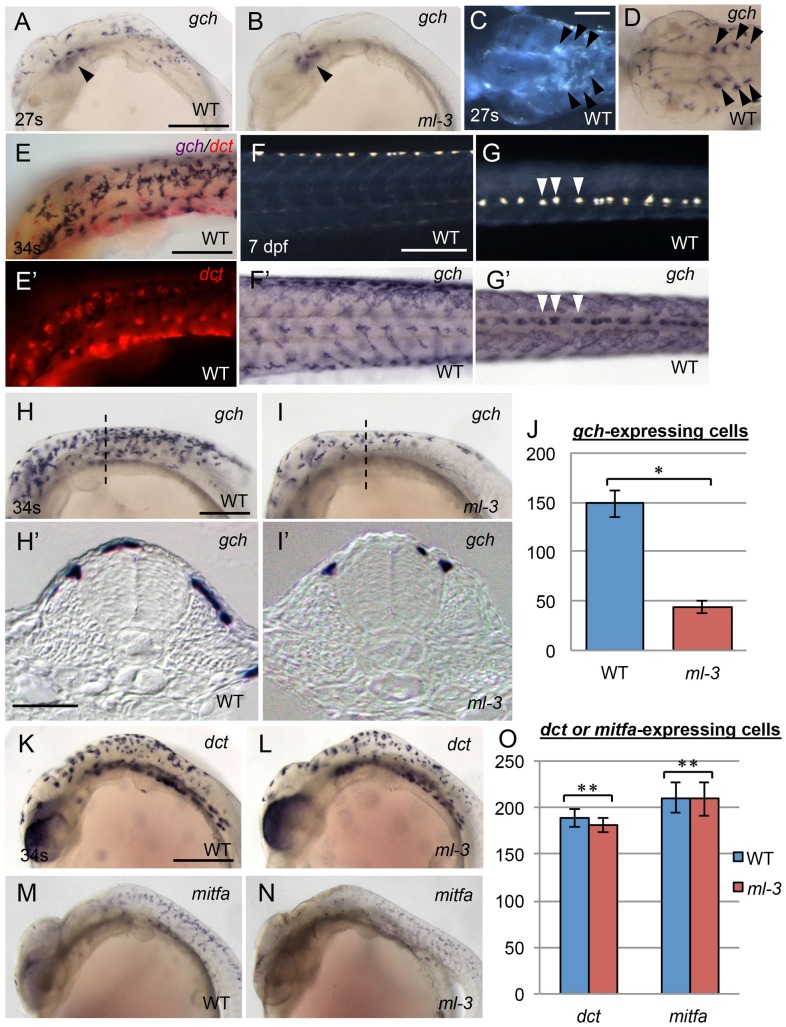
Embryonic pigment cell precursor formation in *ml-3* mutants. (A, B, D, F′, G′, H, I), *gch*. (K, L), *dct*.(M, N), *mitfa.* (E, E′) *gch* (blue) and *dct* (red). (A, C–H, K, M) WT. (B, I, L, N) *ml-3* mutant. (A, B, E, F, H, I, K–N) Lateral views. (C, D, G) Dorsal views. (H′, I′) Transverse sections. (C, F, G) Pre-fixed samples in darkfield. (A–D) 27 somite stage (27 s, 61 hpf). (E, H, I, K–N) 34 somite stage (34 s, 74 hpf). (F, F′, G, G′) 7 dpf. (A–I) ISH analyses show *gch* expression in WT xanthophores and leucophores. Whereas *gch*-expressing cells are found over the lateral and anterior trunk surface and the head in WT at 27 s (A) as well as in ventral head leucophores (black arrowheads, C, D), *ml-3* embryos have *gch* expression only in ventral head leucophores (black arrowheads, B). The embryo at 34 s is co-stained with *gch* riboprobe (purple) and *dct* riboprobe (red) (E, E′). *gch* signals show no overlap with *dct* signals. At 7 dpf, *gch* expression is detected on the surface of the whole length of trunk in WT (F′, G′). The dotted *gch* signals in the dorsal midline coincide with leucophore positions (compare F′ with F and G′ with G, some examples were represented by white arrowhead in G and G′). In WT at 34 s (H), *gch*-expressing cells are spread more posteriorly and scattered over the dorsal trunk surface (H′). In *ml-3* mutants (I), *gch* expression is in fewer cells (J), and these are restricted to the dorsal trunk surface (I′) compared with WT. Transverse histological section from embryos at the level as indicated by dotted line in H and I. (J) Counts of *gch*-expressing cells at 34 s (n = 11 for WT, n = 10 for *ml-3*). The number is significantly fewer in *ml-3* than in WT (*, *p*<0.0001 by Student's *t*-test). (K–N) Melanophore precursors, detected using *dct* (K, L) and *mitfa* (M, N) are not markedly altered in *ml-3* (L, N) as compared with WT (K, M). (O) The number of *dct*- or *mitfa*-expressing cells is not significantly different between WT and *ml-3* (*dct*, n = 11 for each group, **, *p*>0.05 by Student's *t*-test; *mitfa*, n = 12 for each group, **, *p*>0.05 by Student's *t*-test). (J, O) Mean (±s.d.) counts are shown as bars in blue (WT) and in red (*ml-3*). Scale bars: (A, F, H, K) 250 µm; (C) 100 µm; (E) 150 µm; (H′) 25 µm.

To address whether *ml-3* mutation affects the specification of the xanthophore and leucophore lineages, we examined the expression of the earliest known markers for pigment cell precursors. GTP cyclohydrolase I (*gch*) converts GTP to dihydroneopterin triphosphate, which is a source material for pteridine pigments in xanthophore precursors [35], [36]. Dihydroneopterin triphosphate is also converted into H_4_biopterin, which functions as a cofactor for eumelanin synthesis in melanophores. Thus *gch* was reported to be expressed in both xanthophore and melanophore lineages in zebrafish [35]. Our in situ hybridization (ISH) analysis in WT embryos showed that *gch* (XM_004085058) was expressed in laterally migrating cells on the trunk surface and the ventral head leucophores at 24–27 somite stage (58–61 hpf) (Figures 2A, 2C, 2D). The *gch*-expressing cells were located on lateral (above somite) and dorsal (above neural tube) trunk surface at 34 somite stage (74 hpf, Figures 2H, 2H′). These cells included both unpigmented and differentiating pigment cells. Unlike in zebrafish, we saw no evidence for a transient overlap of *gch* expression with that the melanophore marker, *dopachrome tautomerase* (*dct*, XM_004081780) [37], indicating that medaka *gch* was not expressed in the melanophore lineage at the stages tested (Figures 2E, 2E′). Furthermore, at 7 dpf, *gch* mRNA was detected in all xanthophores and leucophores (Figures 2F, 2F′, 2G, 2G′). These data show that, in medaka, *gch* expression is a marker for xanthophore and leucophore linages from well before overt differentiation, but is not a marker for the melanophore lineage at these stages.

Similar results were obtained with *xanthine dehydrogenase (xdh*, XM_004066478), which is known as a xanthophore precursor marker in zebrafish (Figures S3A–S3D) [35]. Whereas *xdh* expression is specific to xanthophores in zebrafish, which do not have leucophores, *xdh* was clearly detectable in both xanthophores and leucophores in medaka. These observations are consistent with previous reports showing that medaka xanthophores and leucophores contain pteridines (sepiapterin in xanthophores and drosopterin in leucophores) and that the enzymes encoded by *gch* and *xdh* are required for pteridine synthesis [11]. Having defined *gch* (which was expressed more strongly than *xdh*) as an early marker of xanthophore and leucophore specification, we then used it to assess whether xanthophore specification, as opposed to visible differentiation, failed in *ml-3* mutants.

At 24–27 somite stage (58–61 hpf), the *ml-3* embryos lacked *gch*-expressing cells on the trunk although they had *gch* expression in the ventral head leucophores (Figure 2B). At 34 somite stage (74 hpf), many, but fewer than in WT embryos, *gch*-expressing cells were detected in *ml-3* mutants (Figures 2H–2J). Transverse cross sections of these embryos revealed that, in *ml-3* mutants, *gch*-expressing cells were detected on the dorsal trunk surface, but were fully absent from the lateral trunk surface (Figure 2I′). Differentiating leucophores and xanthophores are localized on the dorsal and lateral trunk surface, respectively, so that our data indicate that specification of the xanthophore lineage fails in *ml-3* mutants. Expression of melanophore precursor markers, *dct* and *mitfa* (KC249980) was indistinguishable in *ml-3* and WT siblings at 34 somite stage (74 hpf) (Figures 2K–2O), further confirming that *ml-3* mutation specifically affects xanthophore/leucophore lineages.

### *ml-3* locus encodes *sox5*

**Figure 3:**
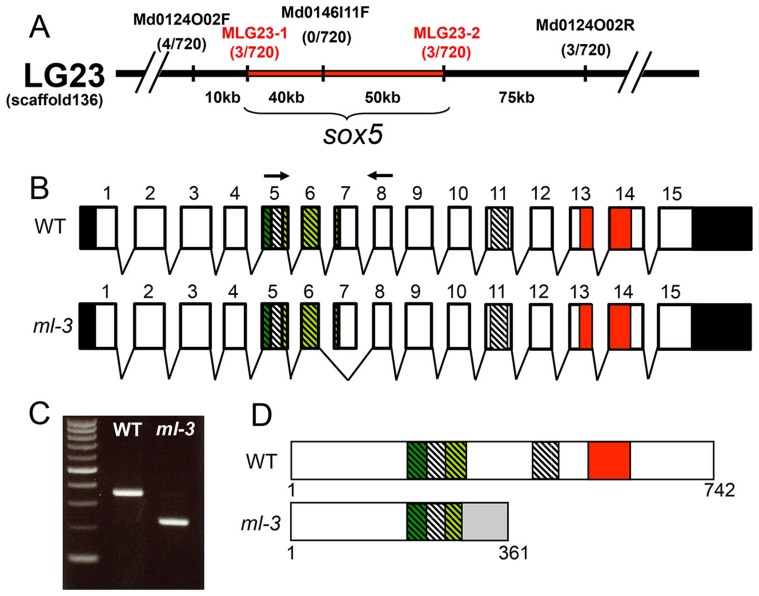
Mapping of the *ml-3* locus. (A) *ml-3* locus was mapped to a 90 kb region (red bar) between MLG23-1 and MLG23-2 on LG23, predicted to contain only one gene, *sox5*. Typing markers are indicated above the map, with the number of recombinants per total 720 haploid genomes examined at each position. (B) Medaka *sox5* comprises 15 exons (upper). Sequencing of cDNAs showed that in *ml-3*, exon 7 is skipped (lower). Boxes represent exons. Angled lines represent introns. The 5′ and 3′ untranslated regions are colored in black. Diagonal stripes and colored regions correspond to regions encoding the protein domains described in (D). Black arrows show positions of the primer set used for RT-PCR in C. (C) RT-PCR detects the skipping of exon 7 in *sox5* mRNA of *ml-3* mutant. (D) WT *sox5* gene encodes 742 amino acid protein consisting of two coiled-coil domains (diagonal stripe) and HMG box (red box). The first coiled-coil domain contains a leucine-zipper (green box) and a glutamine-rich domain (Q box, light green box). In *ml-3*, loss of exon 7 causes a premature stop codon leading to a truncated protein. Gray box represents an altered frame. The resultant truncated protein would lack the second coiled-coil domain and HMG box (lower).

To identify the *ml-3* locus, we carried out positional cloning. The *ml-3* locus was mapped within a 90-kbp region on linkage group 23. *sox5* (AB856413) was the only known gene in this genomic region (Figure 3A). cDNA cloning identified a medaka *sox5* gene that consists of 15 exons with a 2229 bp open reading frame encoding a 742 amino acid protein (Figures 3B, 3D). Medaka Sox5 protein displayed strong similarities to human SOX5 (NP_008871.3) and zebrafish Sox5 (AY730586.1), (66% and 79% amino acid identities, respectively). Like Sox5 in other vertebrate species, medaka Sox5 contained several conserved domains, such as an HMG box DNA-binding domain, a leucine-zipper, a Q-box and two coiled-coil domains (Figure 3D). RT-PCR and sequencing of the *ml-3* mutant cDNA identified a 124-bp deletion corresponding precisely to skipping of exon 7 (Figures 3B, 3C). The *ml-3* mutation introduces a premature stop codon and results in generation of a truncated Sox5 protein lacking the HMG box domain; this is predicted to result in loss-of-function of Sox5 (Figure 3D).

**Figure 4:**
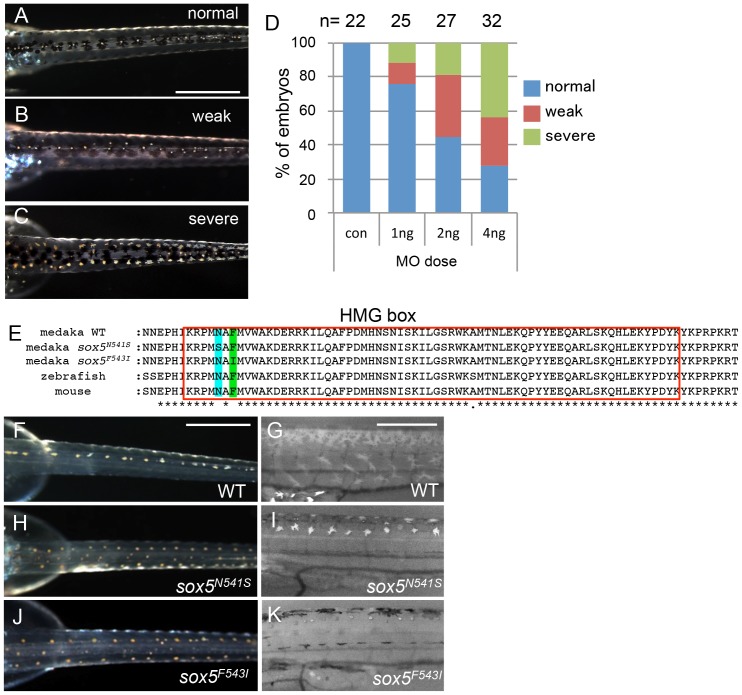
*sox5* morphant and allelic TILLING mutants show the *ml-3* pigment phenotype. (A–D) Injection of *sox5* MO into WT embryo generates *ml-3* mutant phenocopies with increased leucophores in the dorsal trunk compared with embryos injected with the control MO which all show a WT leucophore phenotype (A). The severity of the leucophore phenotype is classified into “weak” (B) and “severe” (C). In “weak” embryos, the majority of leucophores are located along the midline of the trunk, while fewer leucophores are ectopically positioned (B). “Severe” embryos show a phenotype indistinguishable from that of *ml-3* (C). In most cases, general morphology is normal, although about half of *sox5* morphants combined a “severe” pigment phenotype with many ectopic leucophores with gross morphological abnormalities (data not shown). (D) Using this phenotypic classification, we scored the severity of the *ml-3* morphant phenotypes at three different doses (1 ng, 2 ng and 4 ng) and with control MO (con). The number of injected embryos is indicated above the bars (n). (E) Our TILLING screen for *sox5* mutant alleles identified two distinct mutations causing amino acid substitutions at N541S (blue box) or F543I (green box) respectively in the HMG domain (red boxed). (F–K) Homozygotes for the TILLING mutations (*sox5^N541S^* and *sox5^F543I^*) are compared with WT siblings (F, G). These *sox5* mutants exhibit *ml-3* mutant phenocopies as manifested in ectopic and excessive formation of leucophores and complete absence of xanthophores. Leucophores are shown in the darkfield image (H, J). Xanthophores are not detectable under UV light (I, K). Scale bars: 500 µm.

Despite sequencing introns 6 and 7 and exon 7 of the *sox5* gene from *ml-3* mutants, we were unable to identify any candidate mutations that might induce the aberrant splicing of exon 7. To confirm that *sox5* is the causative gene for the *ml-3* phenotype, we carried out knockdown of *sox5* with an antisense morpholino (MO) targeted to the splice donor site of exon 7. The *sox5* morphants exhibited the ectopic formation of leucophores at hatching stage while the control morphants injected with the standard control MO failed to mimic the *ml-3* phenotypes (Figures 4A–4C). As expected, the severity of the morphants' pigment phenotype was dependent on the MO dosage, with higher doses resulting in a higher proportion of embryos closely resembling the *ml-3* leucophore phenotype (Figure 4D). Sox5 morphants also mimicked the xanthophore phenotype of *ml-3* (data not shown). To further confirm that *ml-3* is *sox5*, we carried out TILLING screening for *sox5* mutant alleles in medaka. We successfully isolated two mutant alleles that had a single nucleotide mutation in exon 13 causing an amino acid substitution in the HMG domain of Sox5 (*sox5^N541S^* and *sox5^F543I^*, Figure 4E). The homozygous mutants of both alleles were found to exhibit increased and ectopic formation of leucophores and complete absence of xanthophores, and thus closely phenocopied the *ml-3* mutants (Figures 4F–4K). We conclude that the medaka *sox5* gene is mutated in the *ml-3* mutant.

### *sox5* is expressed in neural crest and xanthophore precursors

**Figure 5:**
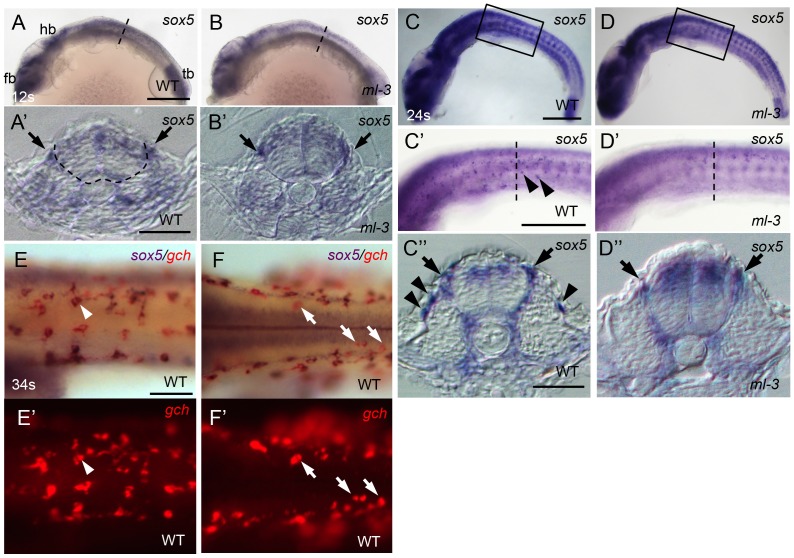
Expression pattern of medaka *sox5* in WT and *ml-3* mutant embryos. (A, C, E, F) WT. (B, D) *ml-3* mutant. (A′, B′, C″, D″) Transverse sections. (E, F) *sox5* (blue) and *gch* (red). (A–E, E′) Lateral views. (F, F′) Dorsal views. (A) In WT embryos at 12 somite stage (12 s, 41 hpf), *sox5* is expressed in premigratory NCCs (see also section in A′, black arrow) as well as in dorsal neural tube and CNS ranging from forebrain (fb) to hindbrain (hb), and in tailbud (tb). The boundary between neural tube and somite is indicated by dotted line. (B) In *ml-3*, the *sox5* expression pattern is not markedly altered. In particular, when observed in section (B′), premigratory NCCs are positive for *sox5*. (C, C′, C″) At 24 somite stage (24 s, 58 hpf), *sox5*-expressing cells are found in dorsal neural tube, premigratory NCCs (arrows in C″) and migrating NCCs between neural tube and somite and lateral trunk surface (black arrowheads in C′, C″) pathways in WT. *sox5*-expressing cells scattered on lateral trunk surface are prominent in WT (C′, C″). (D, D′, D″) In *ml-3*, *sox5* expressing cells are absent from lateral trunk surface (D′, D″), whereas *sox5* expression remains in dorsal neural tube, premigratory NCCs (black arrows) and migrating NCCs between neural tube and somite (D″). Boxed portion in C, D are magnified in C′, D′, respectively. (A′, B′, C″, D″) Transverse histological section from embryos at the level as indicated by dotted line in A, B, C′ and D′. (E, E′) *sox5*-expressing cells on lateral trunk surfaces at 34 somite stage (34 s, 74 hpf) also express *gch*. White arrowhead represents an example of *gch*-positive *sox5*-expressing cell. (F, F′) On dorsal trunk surface, some *sox5*-negative *gch*-positive cells were detected (white arrows). Scale bars: (A, C) 200 µm; (C′) 100 µm; (A′, C″) 20 µm; (E) 50 µm.

Medaka *sox5* mRNA was detected throughout embryonic and early larval stages by RT-PCR (Figure S4). We used whole-mount ISH to determine the expression pattern of *sox5*. In WT embryos, *sox5* mRNA was detected in head and tail bud regions before the onset of somite formation (data not shown). At the 12 somite stage (41 hpf), *sox5* was expressed in the dorsal neural tube and premigratory NCCs (Figures 5A, 5A′). Slightly later *sox5* expression was detected in migrating NCCs ventrally on a pathway between somite and neural tube (medial pathway). At 22–24 somite stage (54–58 hpf), some scattered *sox5*-expressing cells were observed on lateral trunk surface (Figures 5C–5C″). These signals became detectable before the onset of *gch* expression, but, at later stages, overlapped with that of *gch* in differentiating xanthophores on lateral trunk surface; in addition, a few *gch*-expressing *sox5*-negative cells were detectable on dorsal trunk surface (Figures 5E, 5E′, 5F, 5F′). We interpret these data as indicating that *sox5*-expressing cells on lateral trunk surface are differentiating xanthophores (xanthophore precursors), whereas leucophores (dorsal trunk cells) do not express *sox5*. The expression in the xanthophore lineages gradually faded after 4 dpf and became undetectable by 5 dpf. In *ml-3* embryos, the *sox5* expression was not significantly altered in the dorsal neural tube, premigratory NCCs, and the NCCs migrating on the medial pathway. However, the *sox5*-expressing cells on the lateral trunk surface were absent in *ml-3* mutants (Figures 5B, 5B′, 5D–5D″). These results indicate that *sox5* is expressed in the xanthophore precursors and *sox5* is required exclusively for development of xanthophores.

### Sox5 functions cell-autonomously in xanthophore lineage

**Figure 6:**
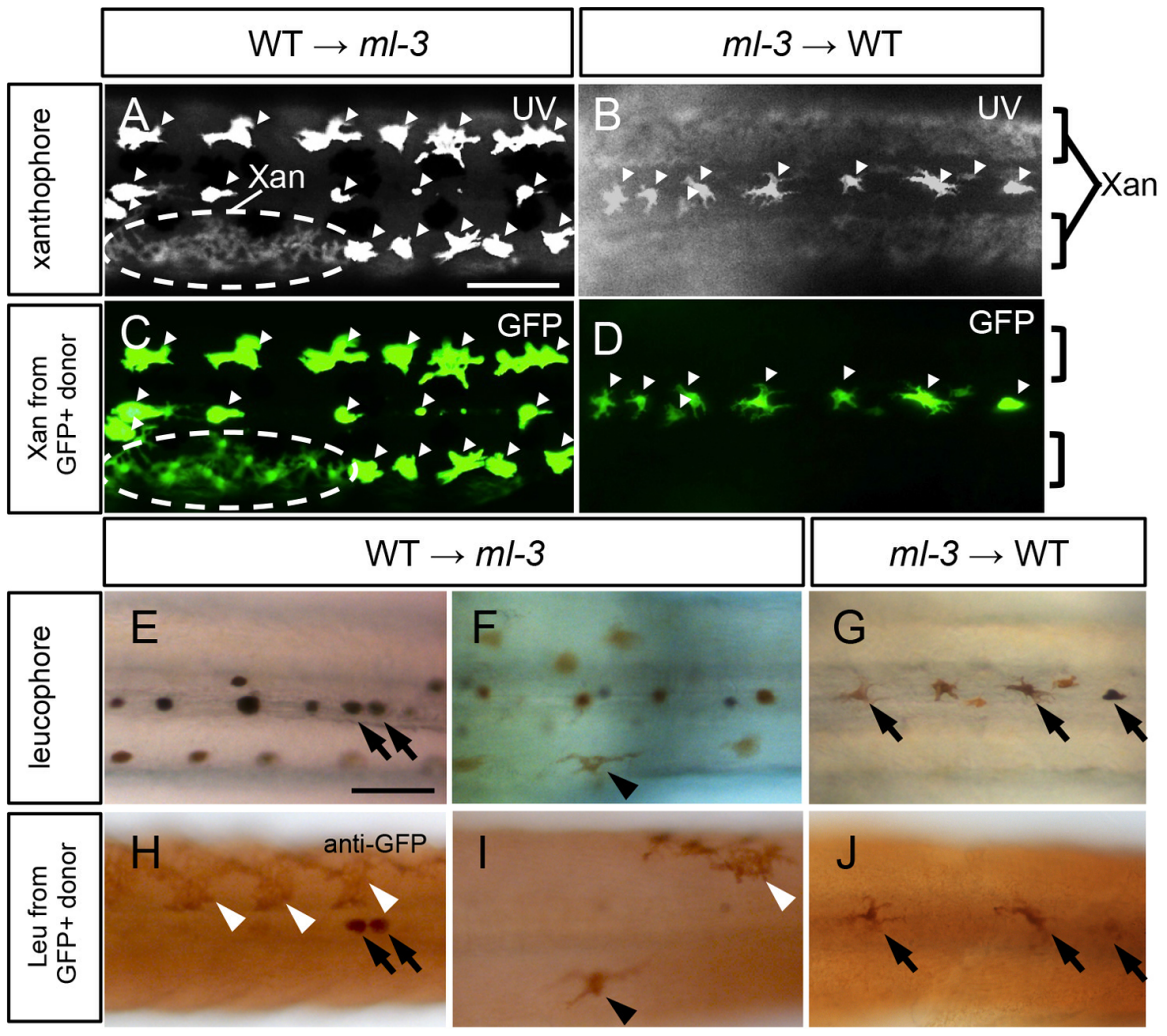
Cell transplantation. (A, C, E, F, H, I) Transplants of WT donor cells into *ml-3* hosts. (B, D, G, J) Transplants of *ml-3* mutant donor cells into WT hosts. (A–D) Note that leucophores are fluorescent through both UV and GFP filters (white arrowheads) and xanthophores are fluorescent through UV filter but not through GFP filter. In both types of transplant experiments, xanthophores and leucophores express GFP when generated from donor cells having *slc2a15b:GFP* transgene, but in leucophores the GFP signal is masked by strong auto-fluorescence of these cells. However, GFP expression is readily detectable by immunostaining (see Figures S5A–S5C). (A, C) In WT→*ml-3* transplants, ectopic leucophores are partially lost and instead a patch of xanthophores (marked with dotted circle) positive for UV (A) and GFP (C) fluorescence can be seen. (B, D) In *ml-3*→WT transplants, leucophores and xanthophores develop and are positioned normally although leucophores derived from donor cells cannot be identified because GFP fluorescence is masked by strong auto-fluorescence. No xanthophores show GFP fluorescence (compare UV image, B and GFP image, D). (E, H) In one of WT→*ml-3* transplants, a few GFP positive leucophores are found along midline (black arrows). (F, I) In another WT→*ml-3* transplants, leucophores are ectopically formed and positive for GFP (black arrowheads). White arrowheads represent donor-derived xanthophores detected by anti-GFP antibody. (G, J) In *ml-3*→WT transplants, GFP-positive leucophores develop and are positioned normally along midline (black arrows). All images are dorsal views at 7 dpf. Xan, xanthophore; Leu; leucophore. Scale bars: 100 µm.

To test the hypothesis that Sox5 functions cell-autonomously in xanthophore specification, we performed cell transplantation between WT and *ml-3* embryos. In this experiment, we used transgenic medaka *Tg(slc2a15b:GFP)*, as a donor, which expresses GFP in both of differentiated xanthophores and leucophores (Figures S5A–S5C). First, WT donor cells from *Tg(slc2a15b:GFP)* embryos were transplanted into *ml-3* host embryos. In some of these WT→*ml-3* transplants, xanthophores were partially restored in patches of the body surface (15/31, Figure 6A, Table S1). All xanthophores in the transplants had GFP signals (15/15, Figure 6C), indicating that only WT donor cells were able to differentiate into xanthophores in *ml-3* hosts. Next, we transplanted *ml-3* donor cells with *slc2a15b:GFP* transgene into WT hosts. Of 25 *ml-3*→WT transplants tested, no embryo had GFP-positive xanthophores (0/25, Figures 6B, 6D, Table S1). We next examined whether *ml-3* donor cells could develop into leucophores in WT hosts. Since GFP fluorescence could not be distinguished from auto-fluorescence in leucophores, we examined GFP expression by immunostaining with diaminobenzidine (DAB). Of 25 *ml-3*→WT transplants, 9 had a few leucophores expressing GFP in the dorsal midline, indicating that *ml-3* donor cells can differentiate into leucophores at the correct position in WT hosts (9/25, Figures 6G, 6J, Table S1). We also tested if WT→*ml-3* transplants had WT donor-derived leucophores. WT→*ml-3* transplants had GFP-positive leucophores at normal and ectopic positions (2/15, Figures 6E, 6F, 6H, 6I, TableS1), suggesting that Sox5 does not control positioning of leucophores cell-autonomously, but rather that absence of xanthophores in *ml-3* mutants may indirectly affect localization of leucophores. To further test this possibility, we performed cell transplantation using *lf-2* mutants, which completely lack xanthophores and leucophores. Of 12 *ml-3*→*lf-2* transplants, 3 had a few ectopic leucophores and no xanthophore (Figures S6A, S6B). This result together with that from *ml-3*→WT transplants supports the idea that Sox5 controls leucophore positioning indirectly by regulating specification of the xanthophore lineage. Considered together with the *sox5* expression in xanthophore precursors, our data indicate that Sox5 controls specification of xanthophores cell-autonomously.

### Role of Sox5 and Pax7a in xanthophore specification

**Figure 7:**
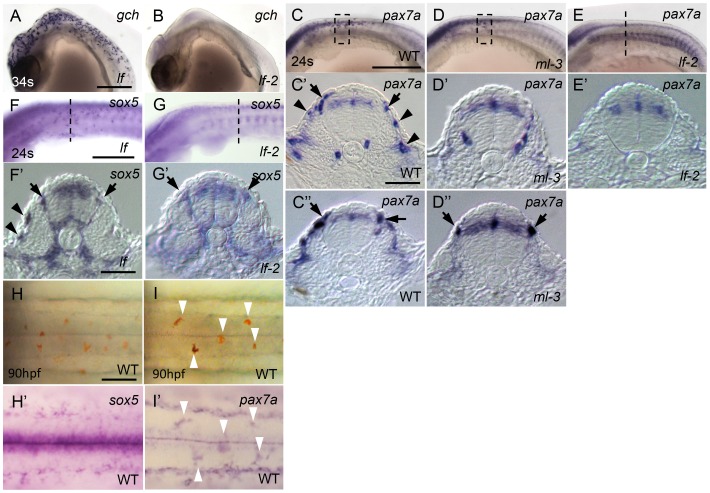
Dual expression of *sox5* and *pax7a* is required for xanthophore development. (A, B) *gch.* (C–E, I′) *pax7a.* (F, G, H′) *sox5.* (A, F) *lf* mutant. (B, E, G) *lf-2* mutant. (C, H, I) WT. (D) *ml-3* mutant. (A–G) Lateral views. (H, I) Dorsal views. (C′, C″, D′, D″, E′, F′, G′) Transverse section. (A, B) *gch* expression in *lf* and *lf-2* at 34 somite stage (34 s, 74 hpf). *gch* is normally expressed in *lf* (A), whereas in *lf-2 gch*-expressing cells are completely absent (B). (C–E) *pax7a* expression in WT, *ml-3* and *lf-2* at 24 somite stage (24 s, 58 hpf). In WT, *pax7a* is expressed in dorsal neural tube, premigratory NCCs (black arrows) in migratory NCCs between neural tube and somite and on lateral trunk surface (black arrowheads, C–C″). In *ml-3*, *pax7a*-expressing cells are lost from lateral trunk surface (D–D″). In *lf-2*, *pax7a* expression in premigratory and migratory NCCs was absent (E, E′). (F, G) *sox5* expression in *lf* and *lf-2* at 24 s. *sox5* expression is normal in *lf* being detected in dorsal neural tube, premigratory NCCs (black arrows) and migrating NCCs between neural tube and somite and on lateral trunk surface (black arrowheads) as in WT (F, F′). In *lf-2*, premigratory NCC expression remains detectable, but *sox5*-expressing cells are lost from lateral trunk surface (G, G′). (E′–G′) The histological section from embryos at the level as indicated by dotted line in E, F and G. (C′, C″, D′, D″) Transverse sections from embryos were selected from the region boxed by dotted line in C and D. (H, I) Expression of *sox5* and *pax7a* on dorsal trunk surface in WT at 90 hpf. (H, I) Pre-fixed WT embryos in bright field. The same embryos were processed for *sox5* (H′) and *pax7a* (I′) ISH. Leucophores are positive for *pax7a* expression (I′, compare white arrowhead positions with I) but not for *sox5* expression (H′). Scale bars: (A, C, E) 200 µm; (C′, E′) 20 µm; (G) 50 µm.

To begin to reveal the gene cascade underlying specification of xanthophore and leucophore lineages, we examined *lf* (*slc2a15b*) and *lf-2* (*pax7a*) mutant embryos (Kimura et al, unpublished data), which each lacks discernible xanthophores and leucophores except for occasional escaper leucophores in *lf-2* [30], [31]. *gch* expression was normal in *lf* but was absent in *lf-2* at 34 somite stage (Figures 7A, 7B). This is consistent with our previous findings that *slc2a15b* is required for pigmentation of xanthophores and leucophores, whereas *pax7a* is required for specification of both xanthophore and leucophore lineages. We next examined the expression of *pax7a* (AB827303) in *ml-3* and *lf-2* mutants, and the expression of *sox5* in *lf* and *lf-2* mutants. Similar to *sox5*, *pax7a* was detected in the dorsal neural tube, premigratory NCCs, and migrating NCCs on the trunk surface in WT (Figures 7C–7C″). We found that *pax7a* was expressed in both differentiating xanthophores and leucophores, whereas *sox5* expression was not detected in early leucophores (Figures 7C, 7C′, 7H, 7H′, 7I, 7I′). *pax7a* expression was absent in cells on the lateral trunk surface in *ml-3* mutants (Figures 7D, 7D′), suggesting that the absence of *pax7a* on the trunk surface in *ml-3* mutants reflects the absence of the xanthophore lineage. The *pax7a*-expressing premigratory NCCs were not altered in *ml-3* mutants (Figure 7D″), whereas *lf-2* mutants lack both of *pax7a*-expressing premigratory NCCs and differentiating xanthophores (Figures 7E, 7E′), suggesting that *pax7a*-positive premigratory NCCs are a shared precursor for both xanthophores and leucophores (shared xanthophore-leucophore progenitor).

As expected, *sox5* expression was normal in *lf* mutants but the *sox5* expression in differentiating xanthophores on lateral trunk surface was not detected in *lf-2* mutants (Figures 7F, 7F′, 7G, 7G′), suggesting *sox5*-expressing specified xanthophore precursors did not form in *pax7a* mutants. Although we expected that *sox5*-expressing premigratory NCCs would be absent in *lf-2* (*pax7a*) mutants, *sox5*-expressing cells were observed at the premigratory position in *lf-2*. This is likely because *sox5*-positive premigratory NCCs contain other progenitor cells than the shared xanthophore-leucophore progenitors. In fact, *sox5* was expressed prominently in cells on the medial pathway presumably giving rise to NCC derivatives other than xanthophores.

To test for genetic interaction between *sox5* and *pax7a*, we generated compound mutants for *sox5* and *pax7a* by crossing the *sox5^+/ml-3^;pax7a^+/lf-2^* parents. The progeny could be categorized into three categories based on their leucophore phenotypes: WT (leucophore+, leucophores are positioned in the dorsal midline of the trunk, 128/233), *ml-3* like (leucophore++, excess leucophores are ectopically located on the dorsal trunk surface, 36/233), *lf-2* like (leucophore-, leucophores are completely absent, 69/233); we did not find any progeny showing phenotypes different from these three (Figures S7A–S7C). The ratio WT:*ml-3* like:*lf-2* like of the categorized larvae was approximately 9∶2.5∶4.8 (Figure S7D). According to Mendelian heredity, the expected ratio of WT:*ml-3*:*lf-2*:*ml-3/lf-2* in the progeny is 9∶3∶3∶1. Our data suggest that *sox5/pax7a* double mutants showed the *lf-2* (*pax7a*) type. Consistent with this, we confirmed that some of the *lf-2* like larvae were homozygotes for the *ml-3* mutation (Figure S7E). In *sox5/pax7a* double mutants, the shared xanthophore-leucophore progenitors did not form and therefore both xanthophores and leucophores did not develop. Our findings indicate that *pax7a* is required for development of the shared xanthophore-leucophore progenitors; *sox5* is required for specification of xanthophore lineage from these progenitors.

## Discussion

**Figure 8:**
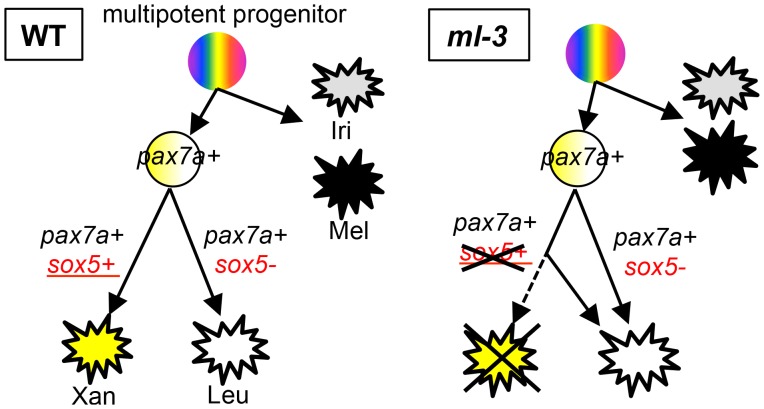
Model for xanthophore and leucophore development from neural crest. We propose that xanthophores and leucophores develop from shared progenitors. Sox5 functions to control fate specification of xanthophores in place of leucophores. In the progenitors, which are positive for *pax7a*, *sox5*-expressing cells are specified to xanthophore fate whereas *pax7a*-expressing *sox5*-negative cells give rise to leucophores. In *ml-3* mutants, loss of functional Sox5 causes a failure of xanthophore specification, resulting in all progenitors becoming specified to leucophore fate. The phenotypes of *lf-2 (pax7a)* and *ml-3 (sox5)*, which are independent of melanophore and iridophore lineages, suggest that xanthophore and leucophore share common progenitors. Mel, melanophore; Iri, iridophore; Xan, xanthophore; Leu, leucophore.

A diversity of pigment cell types in fish provides a unique opportunity to study the mechanisms controlling fate choices in multipotent NCCs. We take advantage of a novel trait in medaka, which have a fourth pigment cell type (leucophores), not seen in most fish species, to uncover the genetic basis for the greater complexity of fate choices. Our combination of studies using positional cloning, gene silencing and TILLING mutant screening have identified medaka *sox5* as the gene responsible for the *ml-3* mutant, which has no xanthophores but excess leucophores in the embryo/larva. Detailed observation of pigment cell phenotypes in *sox5*-deficient mutants revealed that the increase in leucophores and the decrease in xanthophores are *sox5* dosage dependent. Our previous study revealed that *pax7a* is expressed in NCCs and required for development of both xanthophore and leucophore lineages (Kimura et al, unpublished data). In this study, we have demonstrated that *sox5* is expressed in differentiating xanthophores and required cell-autonomously for development of the xanthophore lineage. Furthermore, the ISH analysis of *pax7a* in *ml-3* and *lf-2* mutants and the phenotype of *sox5/pax7a* double mutants suggest that *pax7a* is required for development of the shared xanthophore-leucophore progenitors from NCCs; *sox5* then functions as a molecular switch for specification of xanthophores and leucophores from the progenitors, in which *sox5*-positive and *sox5*-negative cells become specified as and differentiate into xanthophores and leucophores, respectively (Figure 8).

It might be expected that loss of Sox5 activity does not affect total cell number of xanthophores and leucophores if Sox5 determines a fate choice of leucophores versus xanthophores. Our data, however, showed that *gch*-expressing cells, which include both differentiating xanthophores and leucophores, significantly reduced in *ml-3* mutants. Different proliferation rate of xanthophores and leucophores could explain the data. There are fewer leucophores than xanthophores in WT, suggesting that leucophores proliferate less than xanthophores (Figures 1K, 1L). Consistent with this, the *pax7a*-positive progenitors on the dorsal surface were not affected initially (at 18 somite stage) but later during development become significantly reduced in *ml-3* compared to WT (Figures S8A–S8C).

In fish, the three or four pigment cell types are generated from multipotent NCCs, most likely through a progressive restriction mechanism. In zebrafish embryos, *sox10* is required for specification of all non-ectomesenchymal NCC fates, so that loss of Sox10 function disturbs development not only of melanophores but also of xanthophores, iridophores and neural derivatives [38], [39]. Studies in zebrafish show that melanophore/iridophore progenitors are generated from *sox10*-positive NCCs. They initially all express *mitfa*, which is maintained in those that commit to melanophores fate [40]. In the case of iridophore specification, Foxd3 expression promotes iridophore cell fate by repressing *mitfa* expression. A previous study investigating zebrafish *shady (ltk)* mutants showed that the receptor tyrosine kinase leukocyte tyrosine kinase (Ltk) is necessary for iridophore specification [41]. Recently, Lopes et al. also provided evidence that *sox10*-positive*/ltk*-positive cells are likely multipotent progenitors for pigment cells, including melanophores and iridophores [41]. Some of *sox10*-positive*/ltk*-positive cells maintain *ltk* expression and eventually give rise to iridophores. Thus, in zebrafish, it appears that melanophore and iridophore lineages both diverge from a common progenitor cell. Consistent with these insights, *ml-3* mutants have no melanophore nor iridophore phenotype, indicating that, in medaka, the xanthophore/leucophore lineage is specified independently from the melanophore/iridophore lineage.

The leucophore is poorly characterized partly because it has been described in only a few fish species including medaka, but not zebrafish [6]–[8]. Leucophores have been considered to be closely related to iridophores as the hue of both cell types is generated from light reflection due to intracellular purines, uric acid in leucophores and guanine in iridophores [42]. Furthermore, in medaka embryos, leucophores are positioned along the dorsal midline of the trunk and associated with melanophores in a similar manner to that of iridophores in zebrafish embryos [4], [33]. However, xanthophores and leucophores commonly contain pteridine-related substance and our in situ studies revealed that *gch* and *xdh*, which are associated with pteridine synthesis pathway, are expressed in both lineages. Medaka mutants *lf* (*slc2a15b*) and *wl* (*slc2a11b*) affect coloration of both xanthophore and leucophore lineages; Slc2a15b and Slc2a11b functions in the synthesis of pteridines in these pigment cells (Kimura et al, unpublished data). These biochemical traits of xanthophore and leucophore lineages imply a close relationship between xanthophore and leucophore lineages. Although purines are used for reflection of light in both leucophores and iridophores, we speculate that the purine-dependent system may have been co-opted from iridophores when leucophores diverged from the xanthophore lineage during evolution.

Sox5 belongs to the SoxD subgroup together with Sox6 and Sox13 [43]. SoxD proteins are unique in that they have no known transactivation or transrepression domain, despite their transcriptional regulatory actions. Thus, interaction with other transcription factor seems to be important for SoxD to exert its function. Several transcription factors have been suggested to interact with SoxD. In particular, Sox5 and Sox6 cooperate with Sox9 in transactivating chondrocyte-specific genes [44]. In mouse melanoblasts, Sox5 competes against Sox10 for DNA binding sequences and thus antagonistically modulates Sox10 activity to regulate transcription of *mitf* and *dct* [45]. Assuming that Sox10 is generally involved in development of all pigment cell lineages in fish, antagonistic activity of Sox5 against Sox10 may contribute to promote xanthophore lineage in medaka. Previous reports suggest that the interaction between Sox and Pax is important for pigment cell development in mammals. In melanocyte development, Pax3 cooperates with Sox10 in induction of *mitf* expression at early stage [22], [46] but later Pax3 competes with Sox10 to induce the expression of *dct* for terminal differentiation of melanocytes [47]. Pax3 and Pax7 are closely related Pax-family transcription factors with a high degree of sequence homology and a similar genomic organization. These proteins have a similar DNA-binding ability *in vitro* [48]. Given Pax7a functions similar to Pax3 in medaka, Pax7a may function cooperatively with Sox10 during xanthophore/leucophore lineage development in medaka. In this context, Sox5 may act antagonistically against Sox10 interacting with Pax7a within xanthophore progenitor cells in medaka. To address this issue, studies of medaka *sox10* mutants and compound mutants of *sox5*, *sox10*, and *pax7a* are necessary. Further identification of target genes of these transcription factors and master genes for specification and differentiation of xanthophores should help us to understand the molecular mechanism by which Sox5 controls xanthophore specification.

The ectopic localization of leucophores in *ml-3* implies that Sox5 controls NCC migration. However, in our cell transplantation analysis, no *ml-3* mutant donor-derived leucophores were ectopically positioned in WT host embryos. In contrast, we found WT donor-derived leucophores at an ectopic position in *ml-3* host embryo. We noticed that there were no xanthophores around the ectopic leucophores, whereas WT or *ml-3* donor-derived leucophores were observed at the normal positions and laid medially adjacent to xanthophores. Consistent with this, in *ml-3* heterozygotes, excess leucophores are formed at the normal position in the presence of reduced but numerous xanthophores. It has been suggested that the normal pigment pattern formation requires the interactions between distinct pigment cell types [4], [49], [50]. For stripe formation in adult zebrafish, the repulsive behavior between melanophores and xanthophores is suggested to be involved in segregation of the two cell types in the skin. Our findings suggest that the presence of xanthophores prevents leucophores from being positioned laterally close to xanthophores and the absence of xanthophores in *ml-3* mutants allows leucophores to scatter more laterally. Supporting this idea, cell transplantation analysis by using *lf-2* mutants as host embryos has shown that *ml-3* donor-derived leucophores are often formed at ectopic positions in the complete absence of xanthophores. These results imply that repulsive interaction between xanthophores and leucophores likely determines the positions of the two pigment cell types in the medaka embryo.

We show that medaka Sox5 functions as a molecular switch in specification of xanthophores versus leucophores. However, it is not clear whether *sox5* is used for specification of xanthophores in other fish species that have only three types of pigment cells, such as zebrafish. If so, repression of the *sox5* expression in the intermediate progenitor might be linked to appearance of leucophore lineage. Future studies on the role of *sox5* gene in pigment cell development of various vertebrate species should shed light on the mechanism of diversification of pigment cell development.

## Materials and Methods

### Ethics statement

This study was performed with the approval of Nagoya University ethics committee and in accordance with local and Japanese ethical guidelines.

### Medaka strains and husbandry

The Nagoya strain of medaka fish, *Oryzias latipes*, having all of the normal four pigment cell types, was used as WT. *ml-3*, identified in Orange-red stocks (devoid of visible melanophores) by Hideo Tomita [30], was crossed with Nagoya several times to equip the mutant with melanophore prior to experimental use. HNI-I inbred strain (HNI-I/NAGOYA inbred substrain, which was obtained after brother-sister mating of HNI-I inbred strain in more than 15 generations at Nagoya University) was used for crossing in genetic mapping. *lf* and *lf-2* have been described [51]. Fish stocks were maintained in 16-L tanks with a water circulating system (Meitosuien) at 26°C under a 14 h light/10 h dark cycle. In some experiments, embryos were treated with 0.003% phenylthiourea to block melanin synthesis.

### Observation of pigment cells

Embryos and larvae were imaged on a Leica MZ APO or an AxioPlan 2 (Zeiss) microscope using an AxioCam camera (Zeiss). Xanthophores were illuminated with UV light to observe their auto-fluorescence through DAPI filter. Prior to counting melanophore number, embryos were treated with 2 mg/ml epinephrine to aggregate melanin. Significant differences were calculated using Student's *t*-test, one-way ANOVA or two-way ANOVA with GraphPad Prism (version 5.00 for Windows, GraphPad Software).

### Whole-mount in situ hybridization (ISH)

ISH was performed as previously described [52] with modification. A digoxygenin (DIG)- or fluorescein (FITC)-labeled riboprobe was made from a cloned cDNA in pDrive (Qiagen), pCRII-TOPO (Invitrogen) or pGEM-T easy (Promega) by using SP6 or T7 polymerase after restriction enzyme digestion. The brief procedure is as follows. Fixed embryos were treated with Proteinase K (10 µg/ml) in PBS for 10 min at 30°C. Hybridization was performed at 65°C overnight. Signals were detected with alkaline-phosphate conjugated anti-DIG or anti-FITC Fab fragments (Roche) using NBT/BCIP (Roche) or FastRed (Roche) as a chromogenic substrate. For histology, the specimens were embedded in a Technovit 8100 (Heraeus Kulzer) and sectioned at 8-µm thickness.

### Positional cloning

By crossing F1 generation of *ml-3* and WT HNI-I hybrids, 360 F2 offspring displaying the *ml-3* phenotype were collected and subjected to bulked segregant analysis with the M-marker system [53]. For further recombination analysis, polymorphic markers were isolated in reference to medaka genome database (http://medakagb.lab.nig.ac.jp/Oryzias_latipes/index.html). Detailed information on the markers used is shown in Table S2.

### Antisense oligonucleotide-mediated knockdown

Antisense morpholino (MO) oligonucleotide (Gene Tools) for *sox5* was designed on the splice donor site of exon 7. The MO sequence is: 5′-GATGCTACTCACTGCATCCGGGCTT-3′. The non-specific standard control MO was used as a negative control. The sequence is 5′-CCTCTTACCTCAGTTACAATTTATA-3′. MO was microinjected in one blastomere of 1-cell stage embryos.

### TILLING mutant screening

TILLING screening was performed as described [54], [55]. Genomic fragment of exon 13 in *sox5* was amplified with primers; 5′-AATCTTCAGTCGTTTGAGTG-3′ (forward) and 5′-TAACGCTTGCTGAGGAAC-3′ (reverse).

### Generation of transgenic fish

The fosmid clone containing *lf/slc2a15b* region (GOLWFno17_n04) was modified (Kimura et al., unpublished data). Exon 1 of *slc2a15b* was replaced by GFP cDNA (slc2a15b:GFP). The fosmid construct slc2a15b:GFP (50 pg) was injected into 1-cell stage medaka embryos to establish a transgenic line *Tg(slc2a15b:GFP)*. Detailed characterization of *Tg(slc2a15b:GFP)* will be published elsewhere.

### Cell transplantation

Transplanting cells from donor to host was done at late blastula stage. Donors, either of WT or of *ml-3*, were given with the transgene *slc2a15b:GFP* by crossing. Donor and host blastulae were dechorionated with hatching enzyme and placed in groove on a 1.5% agarose plate in 3.5 cm plastic dish filled with balanced salt solution containing 20 U/ml penicillin and 100 µg/ml streptomycin. Approximately a hundred donor cells were transplanted with glass microcapillary. Transplants were incubated at 26°C until 7 dpf.

### Immunohistochemistry

Embryos were fixed with 4% paraformaldehyde in PBS overnight at room temperature. The primary antibody (rat anti-GFP IgG, Nacalai tesque or mouse anti-HuC IgG, Santa Cruz Biotechnology) and the secondary antibody (biotin-conjugated anti-rat IgG or biotin-conjugated anti-mouse IgG, VECTOR) were used at a 1∶1000 dilution. Staining was done by using VECTASTAIN ABC kit (VECTOR) with DAB (MUTO PURE CHEMICALS) or Alexa Fluor 488 tyramide (Invitrogen). In the experiment that detects HuC-positive enteric neurons, immnostaining was performed on cryostat sections made from frozen samples mounted in O.C.T. (Sakura Finetek Japan).

### Alcian blue staining

Hatching larvae were fixed with 4% paraformaldehyde overnight. The samples were washed in acid-alcohol (1% HCl, 70% ethanol) and incubated for 2 hours in 0.1% alcian blue in acid-alcohol, thereafter washed in acid-alcohol and stored in 90% glycerol.

## Supporting Information

Figure S1 Formation of peripheral neurons and craniofacial cartilage in medaka *ml-3* mutants. (A, C, E, G, I) WT. (B, D, F, H, J) *ml-3* mutant. (A, B) Dorsal root ganglions (DRGs), 5 dpf. (C, D) Enteric neurons, 10 dpf. (E–J) Craniofacial cartilages, 9 dpf. (E, F) Lateral views. (G, H) Dorsal views. (I, J) Ventral views. (A, B) Immunohistochemistry with anti-HuC antibody reveals a normal segmental pattern of DRGs (white arrowheads) through the trunk in *ml-3* (B) as compared with WT (A) at 5 dpf. The number of DRGs is indistinguishable between WT and *ml-3* (mean±s.d., 59.2±1.9, n = 10 in WT; 59.3±2.4; n = 6 in *ml-3*; Student's *t*-test, *p*>0.05). (C, D) Transverse sections of the trunk are stained with anti-HuC antibody. *ml-3* larva (D) shows enteric neurons around the gut whose numbers per section are comparable to those in WT (C) (mean±s.d., 8.58±0.33, n = 4 for WT; 8.91±0.27, n = 4 for *ml-3*; Student's *t*-test, *p*>0.05). Means of HuC-positive enteric neurons per section were calculated by counting on sequential 15 sections for each sample. (E–J) The structures of craniofacial skeleton at 9 dpf appear to be normal in *ml-3* as is seen by alcian blue staining. Scale bars: (A) 100 µm; (C) 25 µm; (E) 150 µm.
(TIF)

Figure S2 Embryonic pigment patterns. (A, B) 4 dpf. (C, D, M, N) 7 dpf. (E–L) 5 dpf. (A, C, E, H, K, M) WT. (B, D, G, J, L, N) *ml-3* homozygotes (*ml-3*). (F, I) *ml-3* heterozygotes (+/*ml-3*). (A–G, K–N) Dorsal views. (H–J) Lateral views. (H–J) UV light. (A, C) In WT, leucophores first appear along the dorsal midline at 3.5–4 dpf (A, white arrowheads). By 7 dpf, leucophore become aligned on the midline associated with melanophores (C). (B, D) In *ml-3* mutants, excess leucophores are already observed at 4 dpf (B, white arrowheads) and then form three lines by 7 dpf (D). (E, F, G) In *ml-3* heterozygotes (F), like in WT (E), leucophores are located in the dorsal midline whereas in *ml-3* homozygotes leucophores are scattered over the dorsal trunk (G). The leucophore number in *ml-3* heterozygotes is significantly larger than that in WT and significantly smaller than that in *ml-3* homozygotes (Figure 1K). (H, I, J) In *ml-3* heterozygotes (I), like in WT (H), most xanthophores are positioned laterally to leucophores and melanophores. The xanthophore number in *ml-3* heterozygotes is significantly reduced as compared with WT (Figure 1L). No xanthophores are observed on the trunk surface in *ml-3* homozygotes (J). (K–N) Iridophores appear on the yolk sac (white arrows) at 5 dpf in WT (K) and *ml-3* (L), and increase in number at 7 dpf (M, N). (O, P) Mean (±s.d.) counts of melanophores in DS (O) and leucophores in dorsal trunk (P) in WT (blue) and in *ml-3* (red) are plotted against age. (O) Melanophore number in dorsal stripe is indistinguishable between WT and *ml-3* during embryogenesis (n = 10 for each group and time point): two-way ANOVA, *F*(group) = 0.42, df = 1,144, *p*>0.05; *F*(group×time point) = 1.18, df = 7,144, *p*>0.05 and Student's *t*-test for each time point, **, *p*>0.05). (P) Leucophore number in dorsal trunk is significantly larger in *ml-3* than in WT throughout embryonic stages examined (n = 10 for each group and time point): two-way ANOVA, *F*(group) = 3777.85, df = 1,108, *p*<0.0001; *F*(group×time point) = 47.06, df = 5,108, *p*<0.0001 and Student's *t*-test for each time point, *,*p*<0.0001. Xan, xanthophore; Leu; leucophore. Scale bars: (A) 250 µm; (C) 500 µm; (E) 300 µm; (H) 150 µm; (K, M) 200 µm.
(JPG)

Figure S3 Medaka *xdh* expression is restricted to leucophore and xanthophore lineages. (A, D) Dorsal views. (B, C) Lateral views. At 27 somite stage (27 s, 58 hpf), *xdh* is expressed in ventral head leucophores (white arrowheads) (A) and on lateral trunk surface (B). At 34 somite stage (34 s, 74 hpf), *xdh* expression consists of broadly scattered cells on lateral trunk surface (C). At 5 dpf, *xdh* mRNA is detected in differentiated leucophores (black arrows) and xanthophores (black arrowheads) (D). Scale bars: 100 µm.
(TIF)

Figure S4 RT-PCR of *sox5*. The fragment amplified contains the region from start codon to exon 5. *sox5* mRNA was expressed at all stages examined. Medaka *ef1α* is shown as a positive control.
(TIF)

Figure S5 *Tg(slc2a15b:GFP).* (A) Live image. (B) Fluorescent image. (C) Immunostained image. (A–C) All images are at 7 dpf and dorsal views. In *Tg(slc2a15b:GFP)* embryos, GFP signals are observed in xanthophores on lateral trunk surfaces (black arrows) (B). Leucophores in the midline (black arrowheads, A) show strong auto-fluorescence (B). In these leucophores, GFP signals are also detected by immunostaining (C). Scale bar: 100 µm.
(TIF)

Figure S6 *ml-3*→*lf-2* transplants. (A) Live image in darkfield. (B) UV image. (A,B) At 7 dpf and dorsal views. In *ml-3*→*lf-2* transplants, some leucophores were formed at normal positions in the dorsal midline (arrowheads) and at ectopic positions (arrows, A, B), whereas no xanthophores developed (B). Dorsal midline was shown by dotted line. Scale bar: 250 µm.
(TIF)

Figure S7 Epistasis analysis between *sox5* and *pax7a*. (A–C) Hatching stage (9 dpf) and dorsal views. The offspring from *sox5^+/ml-3^* and *pax7a^+/lf-2^* heterozygote intercross could be classified into three categories based on their leucophore phenotype; WT, leucophores positioned in the dorsal midline (A); *ml-3* like, ectopic leucophores bilaterally scattered (B); and *lf-2* like, leucophores completely lost (C). Numbers of each category are shown in pie graph (D). The ratio WT:*ml-3* like:*lf-2* like of the categorized larvae was 9∶2.5∶4.8 (approximately 9∶3∶4). RT-PCR analysis by using the primer set described in Figure 3B revealed that *ml-3* homozygotes (showing just the small sized fragment resulting from loss of exon 7), were included in some of the *lf-2* like embryos (red boxed, E). Scale bar: 500 µm.
(TIF)

Figure S8 Comparison of the numbers of *pax7a*-positive NCCs between WT and *ml-3*. (A, B) At 18 somite stage (18 s, 50 hpf), *pax7a* expression in NCCs is restricted to the premigratory positions in both WT (A) and *ml-3* (B). (C) The *pax7a*-positive NCCs are counted and shown as means in WT (blue) and *ml-3* (red). At 18 s, the counts have no significant difference between WT and *ml-3*. At 22 somite stage (22 s, 54 hpf) and 24 somite stage (24 s, 58 hpf, also see Figures 7C and 7D), the *pax7a*-positive cells are fewer in *ml-3* than in WT. AT 18 s: WT, 8.3±2.2 (mean±s.d., n = 6); *ml-3*, 7.4±2.3 (n = 5) (Student's *t*-test, *p*>0.05). At 22 s: WT, 21.0±0.9 (n = 4); *ml-3*, 11.0±2.1 (n = 4) (Student's *t*-test, *p*<0.0001). At 24 s: WT, 27.4±4.1 (n = 10); *ml-3*, 13.5±1.7 (n = 8) (Student's *t*-test, *p*<0.0001). Scale bar: 200 µm.
(TIF)

Table S1 The ratio of transplants' phenotypes.
(DOCX)

Table S2 The list of typing markers used in positional cloning.
(DOCX)
