# Supplementary figures and images for: Sox5 Functions as a Fate Switch in Medaka Pigment Cell Development
Source: PLoS Genet. 2014 Apr 3;10(4):e1004246. doi: 10.1371/journal.pgen.1004246 (PMC3974636; doi:10.1371/journal.pgen.1004246)

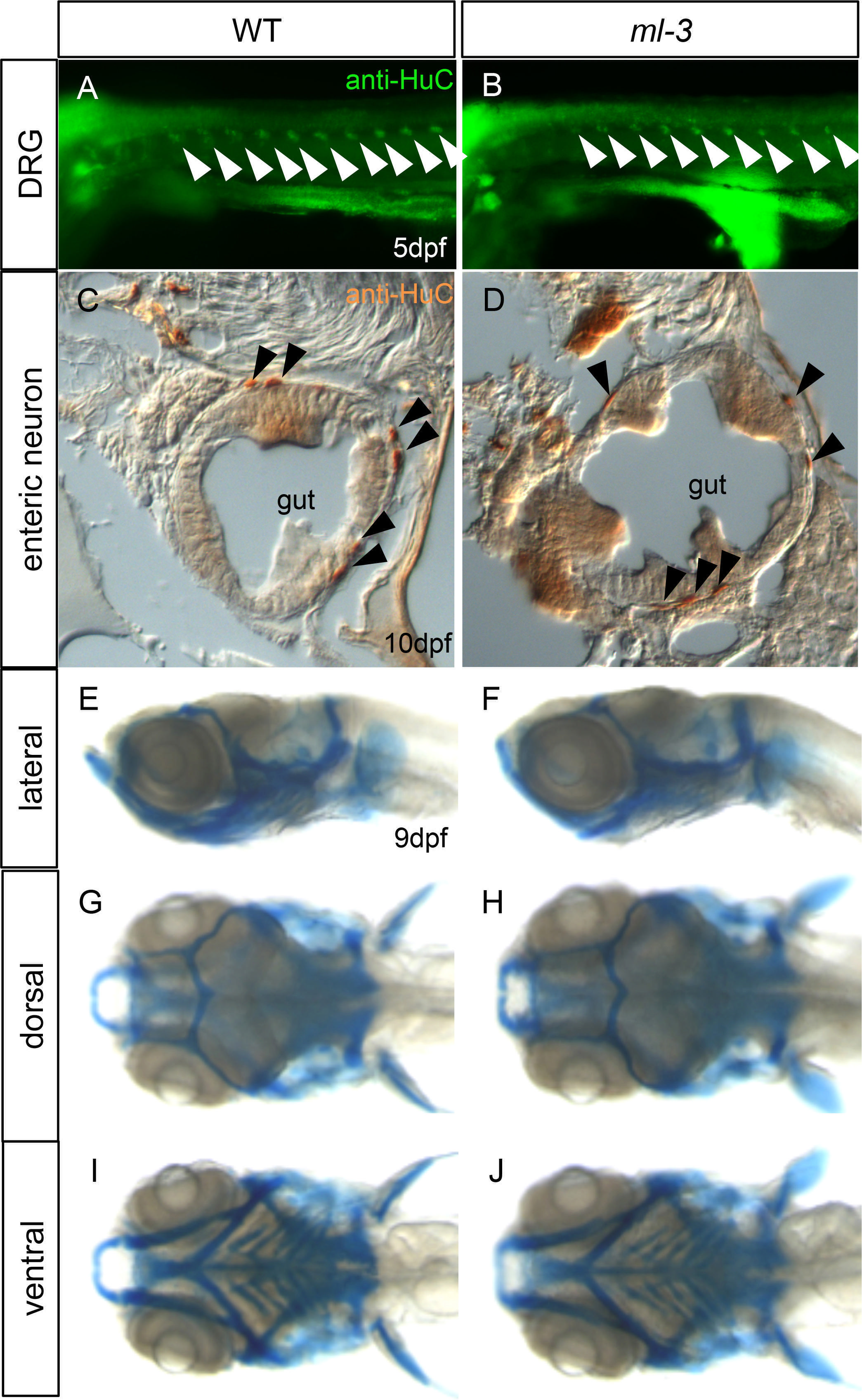

Supplement: Figure S1 — Formation of peripheral neurons and craniofacial cartilage in medaka ml-3 mutants. (A, C, E, G, I) WT. (B, D, F, H, J) ml-3 mutant. (A, B) Dorsal root ganglions (DRGs), 5 dpf. (C, D) Enteric neurons, 10 dpf. (E–J) Craniofacial cartilages, 9 dpf. (E, F) Lateral views. (G, H) Dorsal views. (I, J) Ventral views. (A, B) Immunohistochemistry with anti-HuC antibody reveals a normal segmental pattern of DRGs (white arrowheads) through the trunk in ml-3 (B) as compared with WT (A) at 5 dpf. The number of DRGs is indistinguishable between WT and ml-3 (mean±s.d., 59.2±1.9, n = 10 in WT; 59.3±2.4; n = 6 in ml-3; Student's t-test, p>0.05). (C, D) Transverse sections of the trunk are stained with anti-HuC antibody. ml-3 larva (D) shows enteric neurons around the gut whose numbers per section are comparable to those in WT (C) (mean±s.d., 8.58±0.33, n = 4 for WT; 8.91±0.27, n = 4 for ml-3; Student's t-test, p>0.05). Means of HuC-positive enteric neurons per section were calculated by counting on sequential 15 sections for each sample. (E–J) The structures of craniofacial skeleton at 9 dpf appear to be normal in ml-3 as is seen by alcian blue staining. Scale bars: (A) 100 µm; (C) 25 µm; (E) 150 µm. (TIF) [file pgen.1004246.s001.tif]

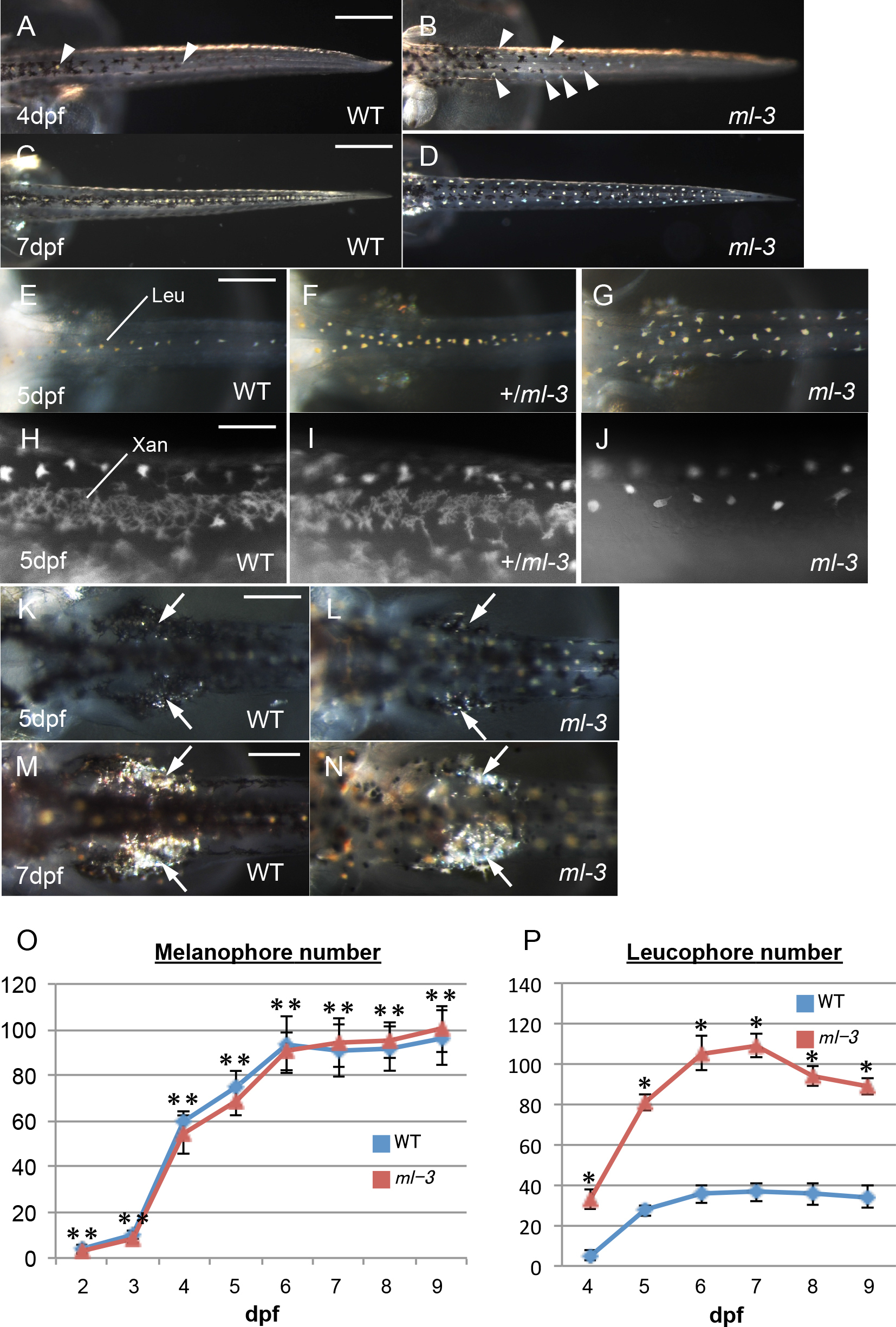

Supplement: Figure S2 — Embryonic pigment patterns. (A, B) 4 dpf. (C, D, M, N) 7 dpf. (E–L) 5 dpf. (A, C, E, H, K, M) WT. (B, D, G, J, L, N) ml-3 homozygotes (ml-3). (F, I) ml-3 heterozygotes (+/ml-3). (A–G, K–N) Dorsal views. (H–J) Lateral views. (H–J) UV light. (A, C) In WT, leucophores first appear along the dorsal midline at 3.5–4 dpf (A, white arrowheads). By 7 dpf, leucophore become aligned on the midline associated with melanophores (C). (B, D) In ml-3 mutants, excess leucophores are already observed at 4 dpf (B, white arrowheads) and then form three lines by 7 dpf (D). (E, F, G) In ml-3 heterozygotes (F), like in WT (E), leucophores are located in the dorsal midline whereas in ml-3 homozygotes leucophores are scattered over the dorsal trunk (G). The leucophore number in ml-3 heterozygotes is significantly larger than that in WT and significantly smaller than that in ml-3 homozygotes (Figure 1K). (H, I, J) In ml-3 heterozygotes (I), like in WT (H), most xanthophores are positioned laterally to leucophores and melanophores. The xanthophore number in ml-3 heterozygotes is significantly reduced as compared with WT (Figure 1L). No xanthophores are observed on the trunk surface in ml-3 homozygotes (J). (K–N) Iridophores appear on the yolk sac (white arrows) at 5 dpf in WT (K) and ml-3 (L), and increase in number at 7 dpf (M, N). (O, P) Mean (±s.d.) counts of melanophores in DS (O) and leucophores in dorsal trunk (P) in WT (blue) and in ml-3 (red) are plotted against age. (O) Melanophore number in dorsal stripe is indistinguishable between WT and ml-3 during embryogenesis (n = 10 for each group and time point): two-way ANOVA, F(group) = 0.42, df = 1,144, p>0.05; F(group×time point) = 1.18, df = 7,144, p>0.05 and Student's t-test for each time point, **, p>0.05). (P) Leucophore number in dorsal trunk is significantly larger in ml-3 than in WT throughout embryonic stages examined (n = 10 for each group and time point): two-way ANOVA, F(group) = 3777.85, df = 1,108, p<0.0001; F(group×time po [file pgen.1004246.s002.jpg]

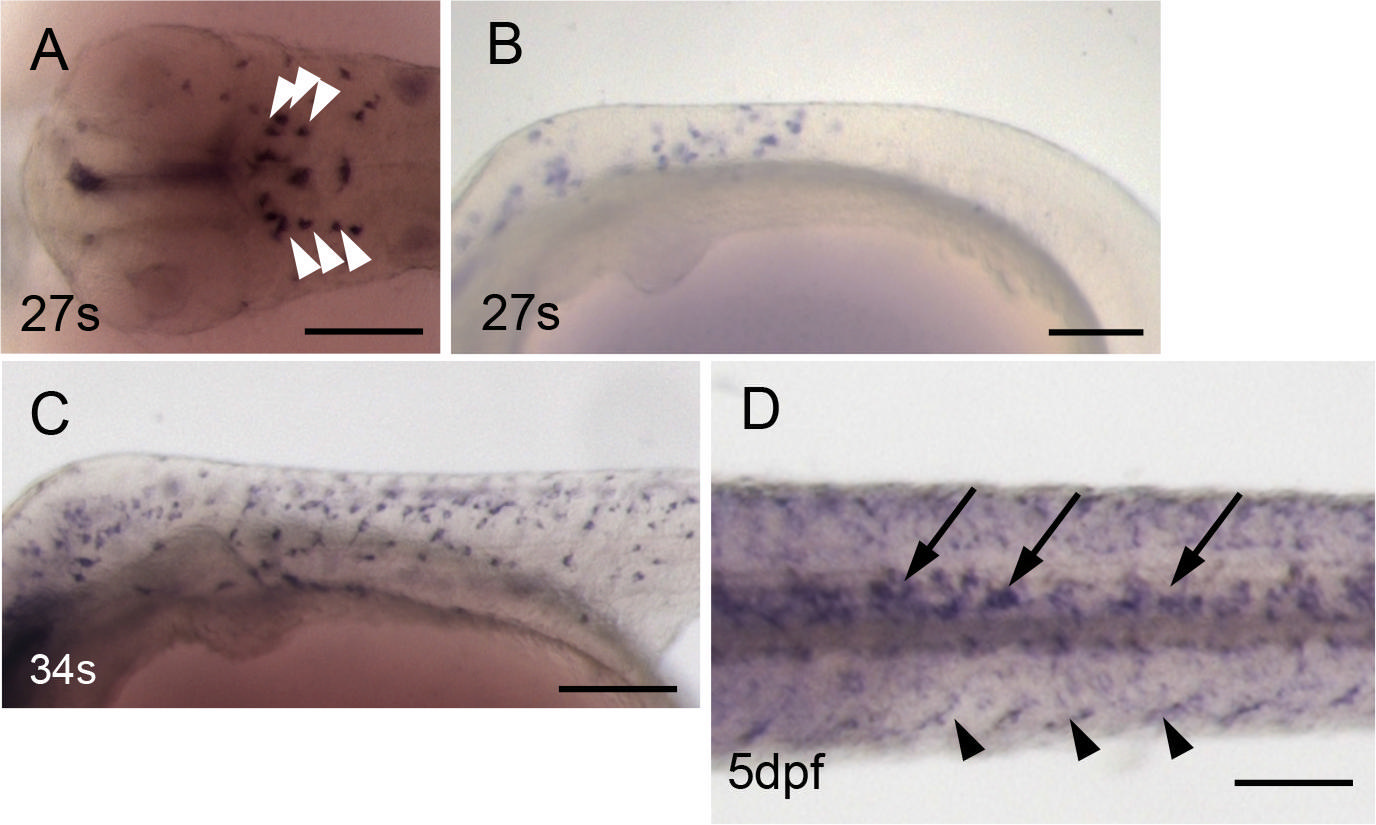

Supplement: Figure S3 — Medaka xdh expression is restricted to leucophore and xanthophore lineages. (A, D) Dorsal views. (B, C) Lateral views. At 27 somite stage (27 s, 58 hpf), xdh is expressed in ventral head leucophores (white arrowheads) (A) and on lateral trunk surface (B). At 34 somite stage (34 s, 74 hpf), xdh expression consists of broadly scattered cells on lateral trunk surface (C). At 5 dpf, xdh mRNA is detected in differentiated leucophores (black arrows) and xanthophores (black arrowheads) (D). Scale bars: 100 µm. (TIF) [file pgen.1004246.s003.tif]

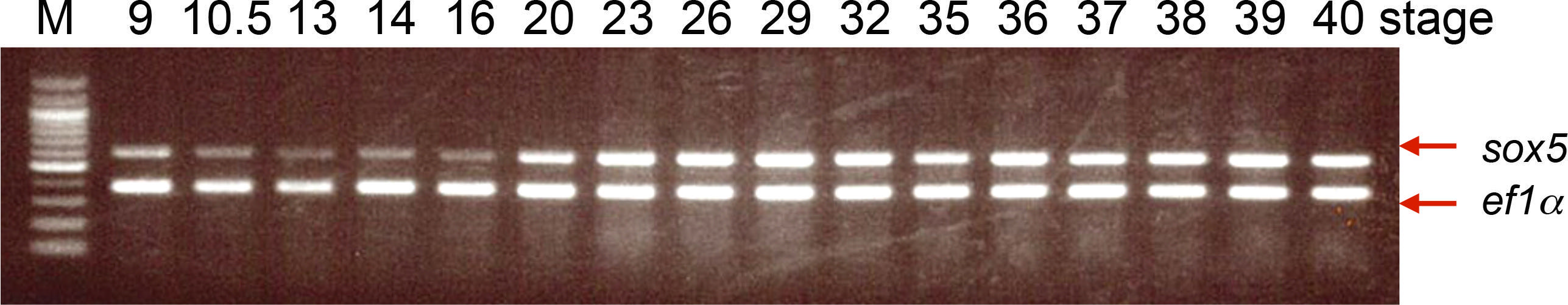

Supplement: Figure S4 — RT-PCR of sox5. The fragment amplified contains the region from start codon to exon 5. sox5 mRNA was expressed at all stages examined. Medaka ef1α is shown as a positive control. (TIF) [file pgen.1004246.s004.tif]

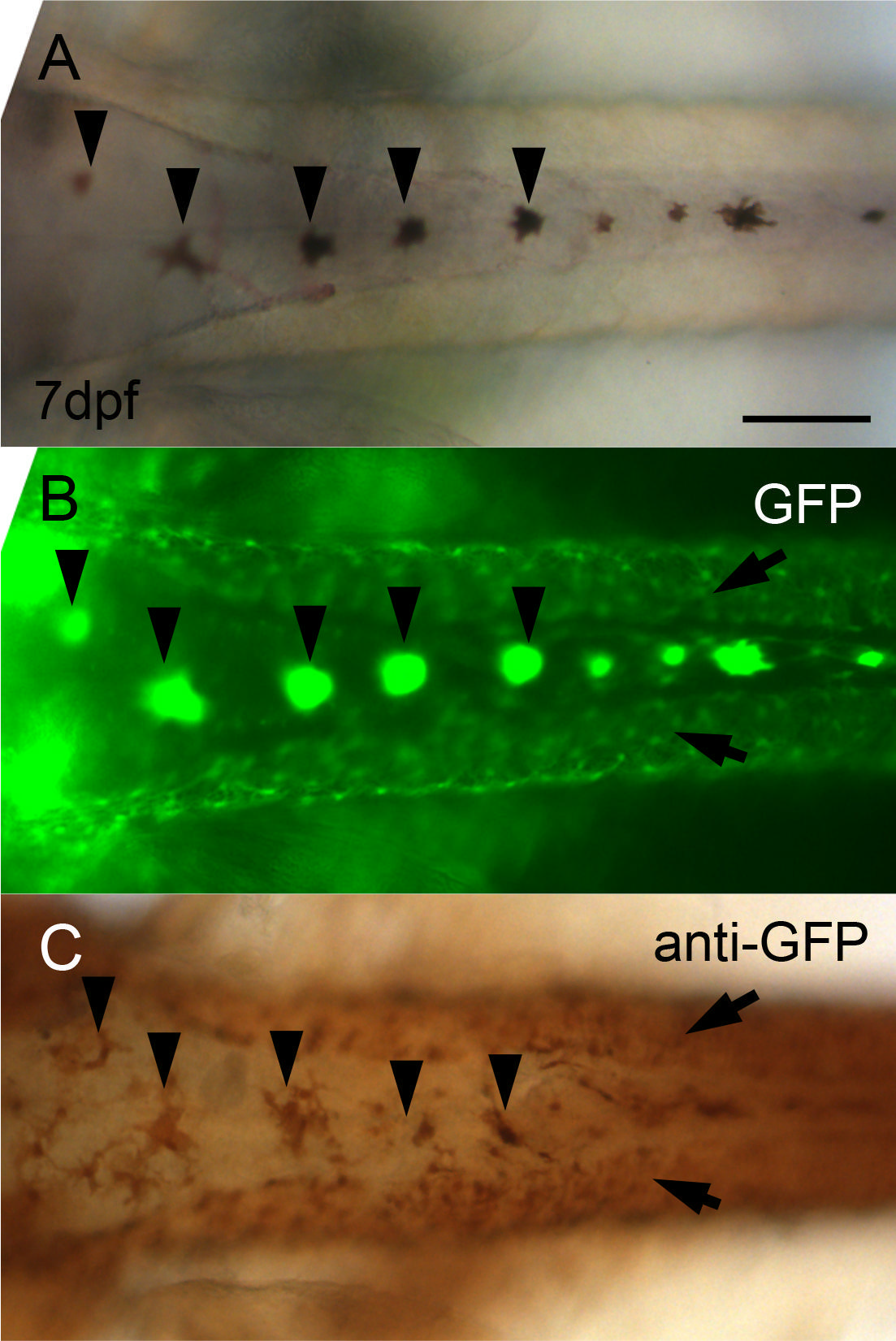

Supplement: Figure S5 — Tg(slc2a15b:GFP). (A) Live image. (B) Fluorescent image. (C) Immunostained image. (A–C) All images are at 7 dpf and dorsal views. In Tg(slc2a15b:GFP) embryos, GFP signals are observed in xanthophores on lateral trunk surfaces (black arrows) (B). Leucophores in the midline (black arrowheads, A) show strong auto-fluorescence (B). In these leucophores, GFP signals are also detected by immunostaining (C). Scale bar: 100 µm. (TIF) [file pgen.1004246.s005.tif]

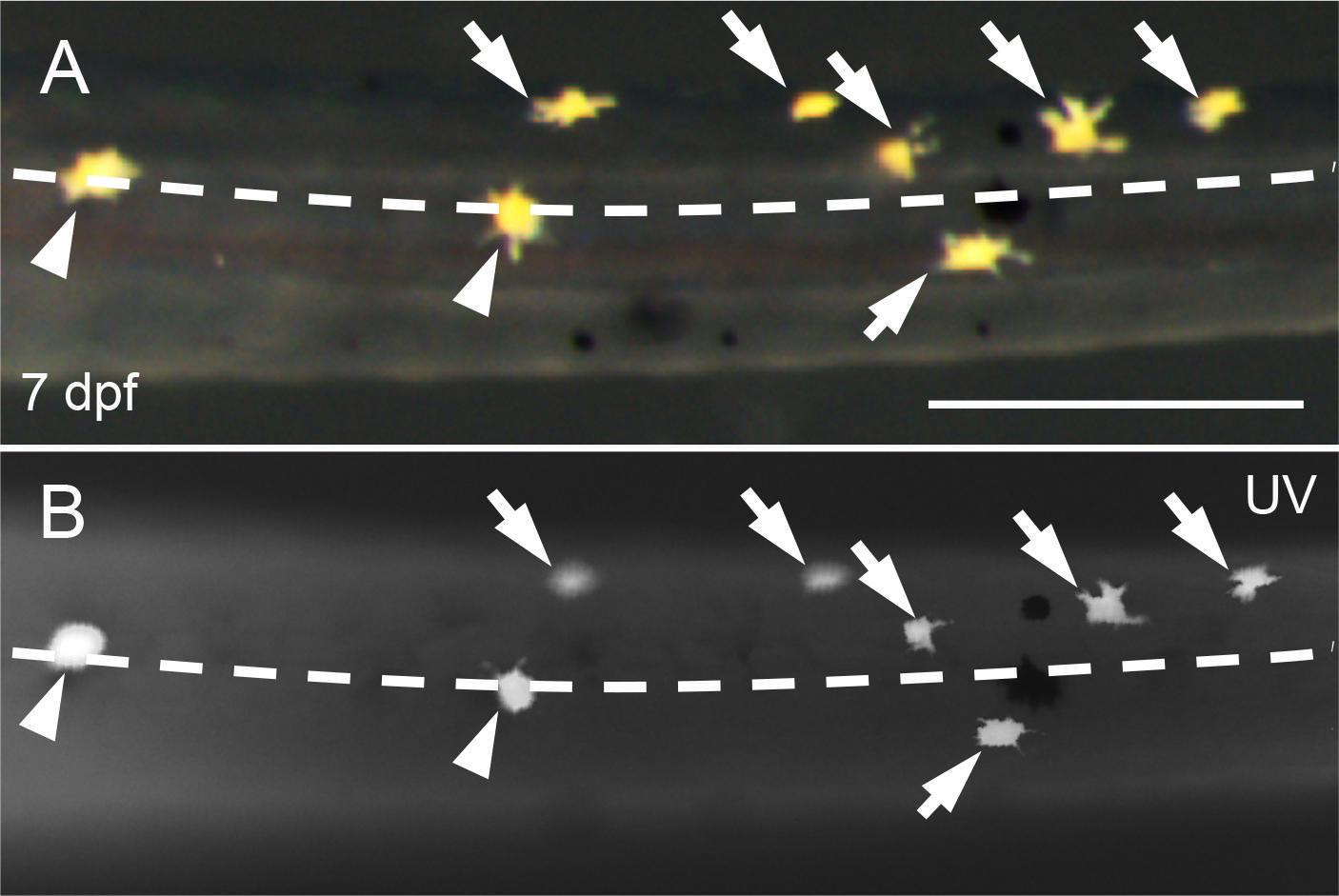

Supplement: Figure S6 — ml-3→lf-2 transplants. (A) Live image in darkfield. (B) UV image. (A,B) At 7 dpf and dorsal views. In ml-3→lf-2 transplants, some leucophores were formed at normal positions in the dorsal midline (arrowheads) and at ectopic positions (arrows, A, B), whereas no xanthophores developed (B). Dorsal midline was shown by dotted line. Scale bar: 250 µm. (TIF) [file pgen.1004246.s006.tif]

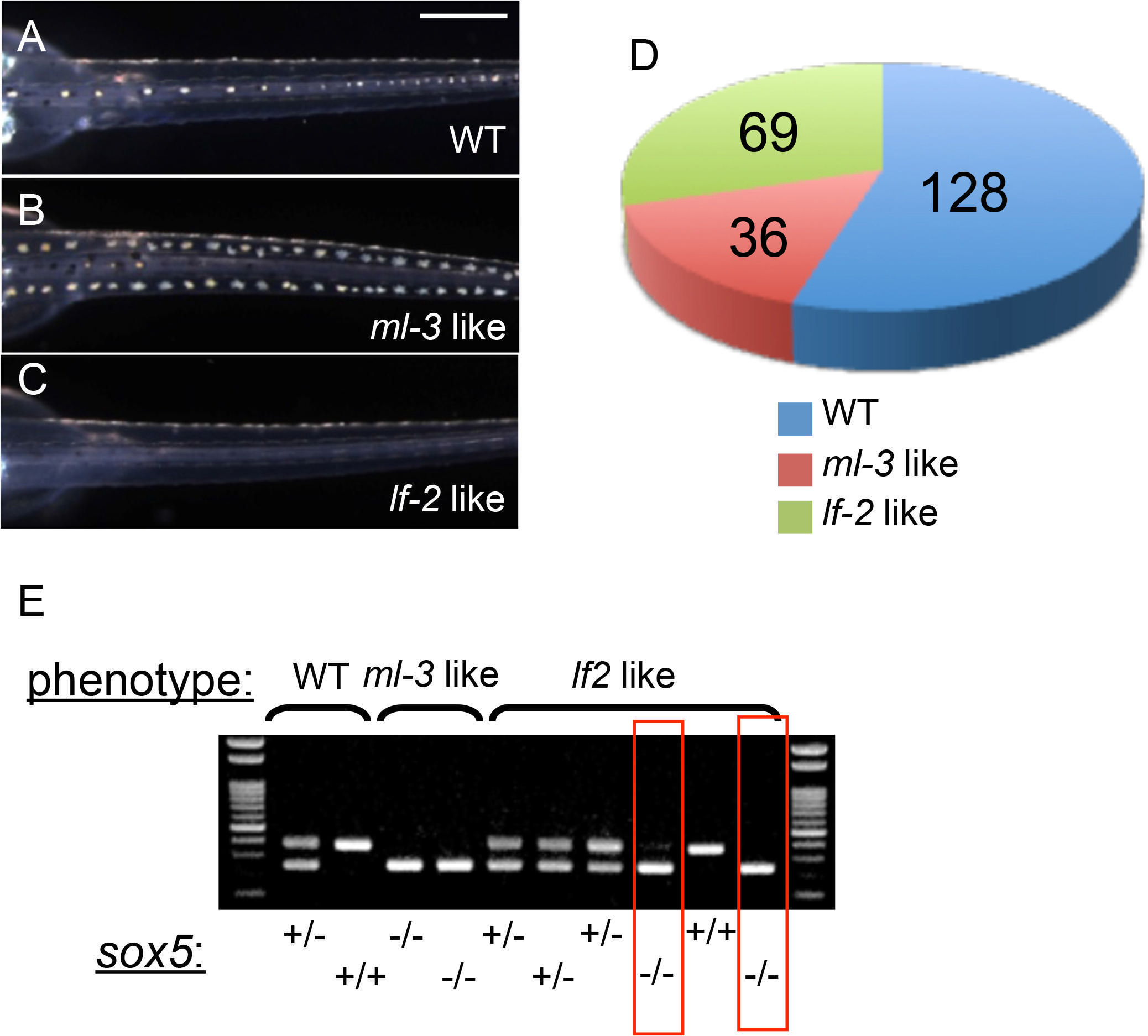

Supplement: Figure S7 — Epistasis analysis between sox5 and pax7a. (A–C) Hatching stage (9 dpf) and dorsal views. The offspring from sox5+/ml-3 and pax7a+/lf-2 heterozygote intercross could be classified into three categories based on their leucophore phenotype; WT, leucophores positioned in the dorsal midline (A); ml-3 like, ectopic leucophores bilaterally scattered (B); and lf-2 like, leucophores completely lost (C). Numbers of each category are shown in pie graph (D). The ratio WT:ml-3 like:lf-2 like of the categorized larvae was 9∶2.5∶4.8 (approximately 9∶3∶4). RT-PCR analysis by using the primer set described in Figure 3B revealed that ml-3 homozygotes (showing just the small sized fragment resulting from loss of exon 7), were included in some of the lf-2 like embryos (red boxed, E). Scale bar: 500 µm. (TIF) [file pgen.1004246.s007.tif]

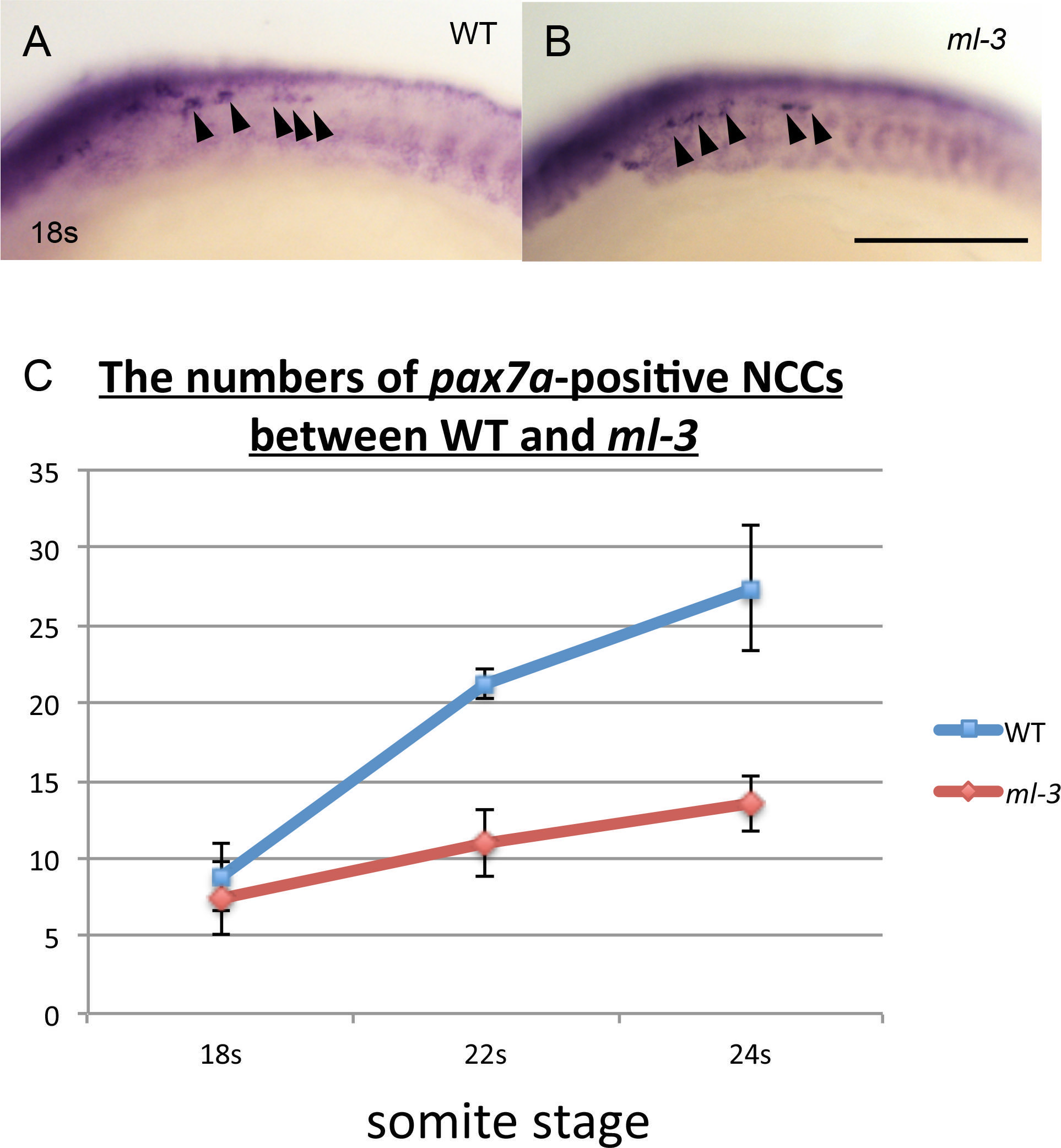

Supplement: Figure S8 — Comparison of the numbers of pax7a-positive NCCs between WT and ml-3. (A, B) At 18 somite stage (18 s, 50 hpf), pax7a expression in NCCs is restricted to the premigratory positions in both WT (A) and ml-3 (B). (C) The pax7a-positive NCCs are counted and shown as means in WT (blue) and ml-3 (red). At 18 s, the counts have no significant difference between WT and ml-3. At 22 somite stage (22 s, 54 hpf) and 24 somite stage (24 s, 58 hpf, also see Figures 7C and 7D), the pax7a-positive cells are fewer in ml-3 than in WT. AT 18 s: WT, 8.3±2.2 (mean±s.d., n = 6); ml-3, 7.4±2.3 (n = 5) (Student's t-test, p>0.05). At 22 s: WT, 21.0±0.9 (n = 4); ml-3, 11.0±2.1 (n = 4) (Student's t-test, p<0.0001). At 24 s: WT, 27.4±4.1 (n = 10); ml-3, 13.5±1.7 (n = 8) (Student's t-test, p<0.0001). Scale bar: 200 µm. (TIF) [file pgen.1004246.s008.tif]
