# Supplementary material for: Sox5 Functions as a Fate Switch in Medaka Pigment Cell Development
Source: PLoS Genet. 2014 Apr 3;10(4):e1004246. doi: 10.1371/journal.pgen.1004246 (PMC3974636; doi:10.1371/journal.pgen.1004246)
Supplement: Table S1 — The ratio of transplants' phenotypes. (DOCX) [file pgen.1004246.s009.docx]

Table S1. The ratio of transplants’ phenotypes.

| WT→*ml-3* | Total | Transplants with  GFP-positive xanthophores | Transplants with GFP-positive leucophores |
| --- | --- | --- | --- |
|  | 31 | 15/31 | 2/15* |
|  |  | Transplants with  *no xanthophores* | Transplants with GFP-positive leucophores |
|  |  | 16/31 | ND** (?/16) |
| *ml-3*→WT | Total | Transplants with  GFP-positive xanthophores | Transplants with  GFP-positive leucophores |
|  | 25 | 0/25 | 0/0 |
|  |  | Transplants with  *GFP-negative xanthophores* | Transplants with  GFP-positive leucophores |
|  |  | 25/25 | 9/25*** |

* One of the two has an ectopic leucophore and the other has endogenously-positioned leucophores.

** Not checked whether leucophores have GFP in the transplants with no xanthophores.

*** All of 9 transplants not having GFP-positive xanthophores have endogenously-positioned leucophores.
