# Supplementary material for: Sox5 Functions as a Fate Switch in Medaka Pigment Cell Development
Source: PLoS Genet. 2014 Apr 3;10(4):e1004246. doi: 10.1371/journal.pgen.1004246 (PMC3974636; doi:10.1371/journal.pgen.1004246)
Supplement: Table S2 — The list of typing markers used in positional cloning. (DOCX) [file pgen.1004246.s010.docx]

Table S2. The list of typing markers used in positional cloning.

|  | forward | reverse | polymorphism |
| --- | --- | --- | --- |
| Mn0124O02F | GCCTTTAGCGTGATCCCTCTGGG | GCAGCAGGCAGGCCTCCTCTTAGG | DdeⅠ |
| MLG23-1 | GCGCACCTGCCTGGTGGACGG | TTATTGGGTGATGTGGTTCTCGTTG | in/del |
| Md0146I11F | CTCGAGAATTCCTCAACAGC | CTCCAAATAGATCAAACAGCCC | in/del |
| MLG23-2 | ATGCTCACTGAGCCTGAGCTACCTC | CCGCCAGAATCTGCATCCAAGACGC | DdeⅠ |
| Md0124O02R | GGTGTGGCCTTTCAACAAGCCC | GTCCTGGTCTTGAATCATC | MseⅠ |

Polymorphisms of PCR fragment were detected by their length (in/del) or RFLP with restriction enzymes (DdeI or MseI).
